# Structures of the human mitochondrial ribosome bound to EF-G1 reveal distinct features of mitochondrial translation elongation

**DOI:** 10.1038/s41467-020-17715-2

**Published:** 2020-07-31

**Authors:** Ravi Kiran Koripella, Manjuli R. Sharma, Kalpana Bhargava, Partha P. Datta, Prem S. Kaushal, Pooja Keshavan, Linda L. Spremulli, Nilesh K. Banavali, Rajendra K. Agrawal

**Affiliations:** 1 Division of Translational Medicine, Wadsworth Center, New York State Department of Health, Empire State Plaza, Albany, NY 12201 USA; 2 Department of Chemistry, Campus Box 3290, University of North Carolina, Chapel Hill, NC USA; 3 Department of Biomedical Sciences, University at Albany, SUNY, Albany, NY 12201-0509 USA; 4 Present Address: High Energy Material Research Lab, Defense Research and Development Organization, Sutarwadi, Pashan, Pune, Maharashtra 411021 India; 5 Present Address: Department of Biological Sciences, Indian Institute of Science Education and Research Kolkata, Mohanpur, West Bengal 741246 India; 6 Present Address: Regional Centre for Biotechnology, 3rd Milestone, Faridabad-Gurgaon Expressway, PO Box # 3, Faridabad, Haryana 121001 India

**Keywords:** Cryoelectron microscopy, Mitochondria, Ribosome

## Abstract

The mammalian mitochondrial ribosome (mitoribosome) and its associated translational factors have evolved to accommodate greater participation of proteins in mitochondrial translation. Here we present the 2.68–3.96 Å cryo-EM structures of the human 55S mitoribosome in complex with the human mitochondrial elongation factor G1 (EF-G1_mt_) in three distinct conformational states, including an intermediate state and a post-translocational state. These structures reveal the role of several mitochondria-specific (mito-specific) mitoribosomal proteins (MRPs) and a mito-specific segment of EF-G1_mt_ in mitochondrial tRNA (tRNA_mt_) translocation. In particular, the mito-specific C-terminal extension in EF-G1_mt_ is directly involved in translocation of the acceptor arm of the A-site tRNA_mt_. In addition to the ratchet-like and independent head-swiveling motions exhibited by the small mitoribosomal subunit, we discover significant conformational changes in MRP mL45 at the nascent polypeptide-exit site within the large mitoribosomal subunit that could be critical for tethering of the elongating mitoribosome onto the inner-mitochondrial membrane.

## Abstract (web summary)

Translation within mitochondria is carried out by specialized mitoribosomes and translational factors. Here the authors describe cryo-EM structures of the human mitochondrial translation elongation factor G1 in complex with human mitoribosomes, revealing distinct mechanism that include conformational changes at the polypeptide exit tunnel.

## Introduction

Mitochondria are thought to have originated through an early endosymbiotic event between an α-protobacterium and a primitive eukaryotic host cell^1^. However, the structural, functional, and compositional organization of the mitochondrial ribosomes (mitoribosome) is dramatically different from its cytoplasmic and bacterial counterparts^2–7^. The ribosomal RNA (rRNA) to ribosomal protein ratio in mammalian mitoribosome (~1:2) is reversed as compared to that in the eubacterial ribosomes (~2:1). The first cryo-EM study of the mammalian mitoribosome identified several unique structural features^5^ in both its subunits: the large 39S subunit (LSU) and the smaller 28S subunit (SSU). Subsequent high-resolution structures^2,4,8,9^ provided molecular description of previously identified features^5^, such as heavily shielded rRNA cores by mitoribosomal proteins (MRPs), the presence of a significantly modified entrance of the mRNA channel and nascent polypeptide-exit tunnel (NPET), and a P-site finger. In addition, the high-resolution structures revealed that one of the mitochondrial tRNAs (tRNAs_mt_) partially substitutes for the role of bacterial 5S rRNA by becoming a structural component of the mammalian mitoribosomal LSU^2,4^.

Similar to bacterial translation, the mechanism of the mammalian mitochondrial translation is roughly divided into four stages: initiation, elongation, termination, and ribosome recycling^10,11^. Each of these steps are facilitated by translational factors that are homologous to their bacterial counterparts but carry mitochondria-specific (mito-specific) segments^10,11^. Biochemical^12–15^ and structural^8,9,16,17^ studies have shown that the mito-specific segments in translational factors play important functions in mitochondrial translation. The distinct structural features in both the mitoribosome and its binding translational factors therefore suggest unique molecular interactions and mechanism during each mitochondrial translation step. The critical step of tRNA and mRNA translocation on the ribosome is promoted by elongation factor-G (EF-G) in eubacteria and homologous EF-2 in eukaryotic cytoplasm. In mammalian mitochondria, there are two isoforms of EF-G_mt_: EF-G1_mt_ and EF-G2_mt_^18,19^. EF-G1_mt_ catalyzes tRNA_mt_ translocation on the mitoribosome, whereas EF-G2_mt_ is involved exclusively in mitoribosome recycling^19^. A mutation in human EF-G1_mt_ leads to fatal hepatoencephalopathy, indicating that this isoform is essential for mitochondrial protein biosynthesis in humans^20,21^. In addition, defects in mitochondrial protein synthesis are associated with numerous human diseases that directly involve mutations in MRPs and tRNAs_mt_^22–24^_._

The bacterial EF-G is composed of five structural domains, namely G (or domain I) and domains II – V^25,26^. The structural and functional aspects of EF-G-catalyzed translocation on bacterial ribosomes have been extensively studied in various functional states, using both cryo-EM^27–33^ and X-ray crystallography^34–38^. Like other translocases, the mammalian EF-G1_mt_ (molecular weight ~80 kDa) is a single polypeptide that possesses mito-specific extensions at both its termini with an additional 47 amino acids (aa), including the signal sequences, as compared to its bacterial homologs^39^. Human EF-G1_mt_ is 751 aa long, where the first 36 residues at the N-terminus constitute the mitochondrial targeting signal^39^, which is cleaved off in the functional form. The functional human EF-G1_mt_ shows ~45% sequence identity with its bacterial counterpart, with a major difference being the presence of an 11 aa mito-specific extension at its C terminus. We have determined near-atomic-resolution cryo-EM structures of the human 55S mitoribosome in complex with the human EF-G1_mt_ to investigate the roles of the mito-specific MRPs and C-terminal extension in EF-G1_mt_ in tRNA_mt_ translocation in mammalian mitochondria. Our study reveals several distinct features, including mito-specific molecular interactions during EF-G1_mt_-mediated tRNA_mt_ translocation on the human mitoribosome. In addition, we identify conformational changes associated with translation elongation at the exit of the NPET within the mitoribosome that could be necessary for facilitating the release of the nascent polypeptide chain through the NPET and anchoring of the mitoribosome on to the inner mitochondrial membrane.

## Results and discussion

### Overall structure of the 55S·EF-G1_mt_ complex

**Fig. 1:**
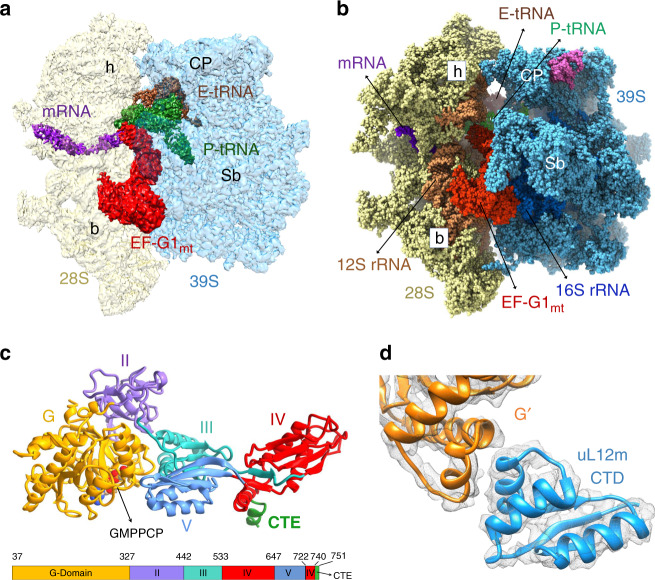
Cryo-EM structure of the human mitochondrial 55S·EF-G1_mt_·GMPPCP Class III complex. **a** Segmented cryo-EM densities, showing 28S (semitransparent yellow), 39S (semitransparent blue), EF-G1_mt_ (red), P-site tRNA_mt_ (green), fragmented E-site tRNA_mt_ (brown), and small segment of mRNA (purple), as viewed from the subunit-subunit interface side. **b** Atomic model of components within the complex, colored as in panel **a**. Landmarks on the 28S subunit: h head, b body. Landmarks on the 39S subunit: CP central protuberance, Sb uL11m stalk base. **c** Structure of EF-G1_mt_ displaying its five domains and the bound GMPPCP molecule, with a color-coded bar diagram depicting domain organization of EF-G1_mt_. **d** Interaction between the C-terminal domain (CTD, blue) of uL12m and G′ subdomain (orange) of EF-G1_mt_.

To obtain the 55S·EF-G1_mt_ complex a non-hydrolysable analog of GTP, GMPPCP, was used to lock EF-G1_mt_ on the mitoribosomes (see Methods). A cryo-EM structure for the 55S·EF-G1_mt_·GMPPCP complex with an overall resolution of 2.7 Å (Supplementary Figs. 1 and 2) was obtained (see Methods). 3D classification of all selected 55S mitoribosome images yielded two major classes that were refined to 2.96 Å and 2.97 Å resolution, respectively, and a minor class that was refined to 3.96 Å resolution (Supplementary Figs. 1 and 2; Supplementary Table 1). All three 55S mitoribosome maps show well-defined densities for EF-G1_mt_, but reveal significant differences in the relative orientation of the 28S subunit with respect to the 39S subunit. The cryo-EM structure with the 28S subunit in its fully rotated state^8^ is referred to as Class I, the structure with the 28S subunit in its unrotated canonical state^2,4^ is referred to as Class III (Fig. 1a, b), and the structure with the 28S subunit an intermediate state between the Class I and Class III conformations is referred to as Class II. Each of these structures shows variable densities for tRNAs_mt_ in the mitoribosomal peptidyl (P) and exit (E) sites (Fig. 1a, b; and Supplementary Notes).

The overall conformation of EF-G1_mt_ on the 55S mitoribosome in all three maps is analogous to the structure of EF-G in the cytoplasmic ribosomal complexes, where the factor has been trapped either with the help of the antibiotic fusidic acid (FA)^27,31,32,35^ or by using a non-hydrolysable GTP analog^29,33,34,36,37^. In addition to determining the structure of complete EF-G1_mt_ with all of its 715 aa residues that fold into five globular domains (Fig. 1c), high-resolution features in our cryo-EM maps (Supplementary Fig. 3) allow us to model 75 rRNA residues and 1,082 aa residues that are absent in the currently available human mitoribosome structure^2^. Furthermore, we identify species-specific structural differences among mammalian MRPs. The EF-G1_mt_ binding stabilizes the flexible C-terminal domain (CTD) of the uL10-L12 stalk^29^ and enables modeling of one copy of the uL12m CTD that interacts with the G′ subdomain of EF-G1_mt_ (Fig. 1d). EF-G1_mt_ induced movement also brings the N-terminal domain (NTD) of uL11m by 5 Å closer to the uL12m-CTD (Supplementary Fig. 4c), thereby enabling the latter to simultaneously interact with the uL11m and the G′ subdomain to form an arc-like structure^27,29^. A direct interaction between uL12m CTD and EF-G1_mt_ suggests uL12m’s role in factor recruitment^40,41^ during mitochondrial translation.

### Interactions of EF-G1_mt_ with the GTPase-activating center of the mitoribosome

**Fig. 2:**
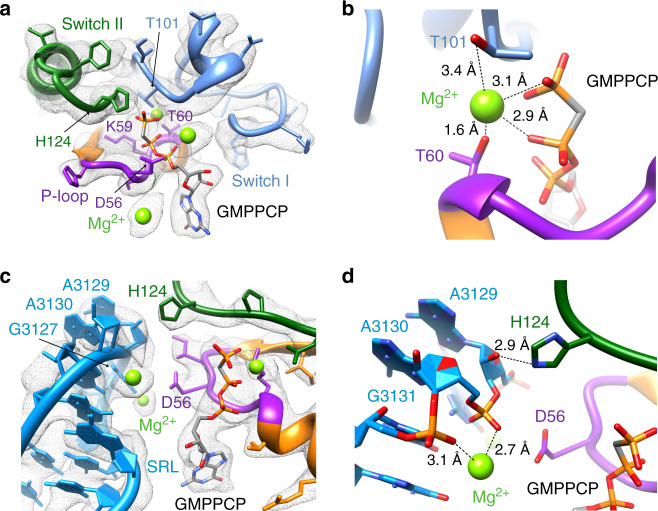
Interactions of GMPPCP and Mg^2+^ within the G domain of EF-G1_mt_. **a** GMPPCP position in the nucleotide-binding pocket through multiple interactions with conserved aa residues of functionally essential elements of the G domain such as Switch I (blue), Switch II (green), and the P-loop (purple). The bound magnesium ions are shown as green spheres. **b** T60 from the P-loop and T101 from the switch I region coordinates a crucial Mg^2+^ ion near GMPPCP. **c** Interactions of H124 (green) and D56 (purple) with the functionally important bases from the α-sarcin-ricin loop (SRL): A3129, A3130, and G3131. **d** H124 is stabilized in its active conformation through interactions with the sugar moiety of a highly conserved residue of the SRL A3129. Phosphate backbones from SRL’s A3130 and A G3131 residues coordinate a Mg^2+^ ion that in turn stabilize D56 in its active conformation. Colors of G domain components in panels **a**–**d** are matched.

Translocation of tRNAs and mRNA is an intrinsic property of the ribosome but binding of EF-G·GTP and subsequent hydrolysis of GTP on EF-G enhances the rate of translocation by several orders of magnitude^42,43^. Using our higher resolution map (Complex III), a complete de novo model of the nucleotide-binding pocket and the interactions of switch I with other domains of EF-G1_mt_ and the adjacent ribosomal components could be constructed. The bound GMPPCP is held in position through a large network of hydrogen bonds and van der Waals interactions with several highly conserved EF-G1_mt_ residues, notably D56 and K59 from the P-loop, T101 from the switch I region, and H124 from the switch II region (Fig. 2a). A crucial Mg^2+^ ion positioned close to γ phosphate of GMPPCP is coordinated by T60 from the P-loop and T101 from the switch I region (Fig. 2b). H124 is known to be essential for catalyzing the hydrolysis of GTP, as mutation of this crucial residue in bacterial EF-Tu severely inhibits its ribosome-stimulated GTP hydrolysis^44,45^. In our maps, the catalytic H124 is oriented towards the γ phosphate of the bound GMPPCP molecule (Fig. 2c), representing an active nucleotide-binding pocket^34,36^ while its analog is pointed in the opposite direction in a GDP-state bacterial post-translocation complex (Supplementary Fig. 5)^35^. The positioning of H124 is stabilized by the universally conserved sarcin-ricin loop (SRL) of the 16S rRNA segment from the 39S LSU, which plays a central role in activating the translational GTPases^34–36^. Binding of G domain adjacent to the SRL closes the nucleotide-binding pocket of EF-G1_mt_ and the base A3129 from the SRL is responsible for stabilizing the current activated conformation of H124 through hydrogen-bonding interactions (Fig. 2c, d). Bases A3130 and G3131 from the SRL are found coordinating a uniquely placed Mg^2+^ ion which in turn is known to stabilize the P-loop D56 in an active conformation (Fig. 2c, d). As observed in bacteria^34,36^, the binding of EF-G1_mt_ to the 55S mitoribosome stabilizes the factor in an active conformation necessary to catalyze the hydrolysis reaction, by ordering the switch I region and positioning of D56 and H124 towards the active site for GTP hydrolysis.

### Interactions of domain IV of EF-G1_mt_ in the A site of the 28S subunit in three conformational states

**Fig. 3:**
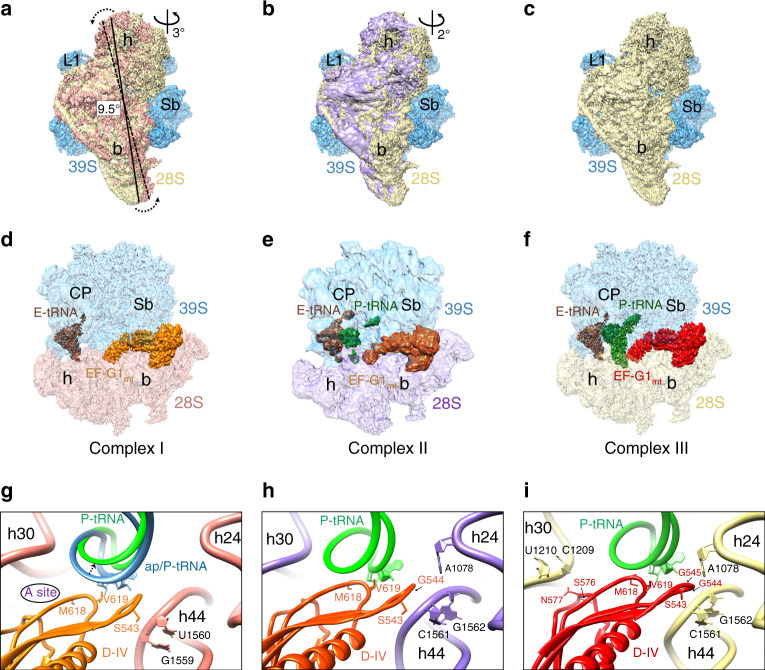
Conformational states of the 28S subunit and interactions of domain IV of EF-G1_mt_ in 55S·EF-G1_mt_·GMPPCP complexes. **a**–**c** Intersubunit rotation and the 28S head swiveling. **a** Superimposition of the cryo-EM maps of the Class I and the Class III complexes revealed an overall 9.5° rotation of the 28S subunit (salmon) in an anti-clockwise direction relative to the 39S subunit (blue) in Class I complex, with an additional rotation of the 28S head domain by ~3° in a roughly orthogonal direction to the direction of the inter-subunit rotation. **b** Similar comparison between the Class II and Class III complexes revealed ~2° rotation of the 28S head in the Class II complex (light purple), without a measurable overall inter-subunit rotation. In all three panels, the 55S mitoribosome is shown from the 28S solvent side. The 28S subunit from Complexes I and II are shown in salmon and purple colors, whereas that from the Class III complex is shown in yellow color. Landmarks for both mitoribosomal subunits in panels **a**–**f** are the same as introduced in Fig. 1, except for L1 stalk (L1). **d**–**f** Segmented densities corresponding to P- (green) and E- (dark brown) site tRNAs_mt_ and EF-G1_mt_ (orange, reddish orange, and red) in complexes I–III are shown, with semitransparent densities for the 28S (bottom) and 39S (top) subunits. **g**–**i** Interactions of EF-G1_mt_ domain IV with the 28S subunit and the P-site tRNA. **g** In the Class I complex, domain IV (orange) is positioned ~10 Å away from the 12S SSU rRNA helices (salmon) h30 and h24. A 6–7 Å shift in the anticodon end of P-tRNA (green) to an ap/P hybrid position (blue) is required to establish contacts with the EF-G1_mt_ domain IV (orange). The A-site location is indicated. **h** Intermediate head swiveled-only state shows limited interactions of domain IV (reddish orange) with the 12S rRNA helices (light purple) h24 and h44. **i** Multiple interactions of domain IV (red) with the 12S rRNA (yellow) and P-site tRNA (green) in the Class III complex.

In all three 55S·EF-G1_mt_ complexes, the EF-G1_mt_ is held in position by interacting with several components of both the large and small mitoribosomal subunits but domain IV of EF-G1_mt_ and mitoribosomal components interact differently among these complexes. In the Class I 55S·EF-G1_mt_ complex, the 28S subunit has undergone a ~9.5° counter-clockwise rotation relative to the 39S subunit (Fig. 3a), similar to previously described ratchet-like inter-subunit reorganization of the bacterial ribosome^30,46^. In addition to this ratchet-like motion, significant head swiveling^32,47^ was also observed in the Class I complex, where the 28S subunit head region has rotated ~3° relative to the body in a roughly orthogonal direction to the ratchet-like motion (Fig. 3a). The Class II complex presents a previously unknown EF-G-bound conformational intermediate, where the head region of the 28S subunit has swiveled as in the ratcheted Class I complex (Fig. 3b), while the conformation of the 28S body region is similar to that in the unrotated Class III complex (Fig. 3c). The presence of a Class I complex-like conformational state with ratcheted and head rotated SSU in a previous EF-G1_mt_ -unbound map^2^ but the absence of such a conformation in our control maps (Supplementary Fig. 6) suggest that the Class I complex is formed either upon binding of EF-G1_mt_ to a subpopulation of mitoribosomes that carries only a single tRNA at the E-site^2,48^ (Fig. 3d), or to a population that carries loosely bound P-site tRNAs that are all translocated to the E-site. Overall, we find that EF-G1_mt_ binding brings a greater proportion of mitoribosomes into unratcheted state, when compared with the distribution in our control population (Supplementary Fig. 6). The Class II complex represents the smallest of the three populations and shows a strong density for the E-site tRNA but a somewhat fragmented tRNA density at the P site (Fig. 3e), whereas the Class III complex shows densities for both P- and E-site tRNAs (Figs. 1a, 3f).

Domain IV of the bacterial EF-G is known to play a crucial role in tRNA and mRNA translocation^27,49^. In all three Classes, Domain IV of the EF-G1_mt_ is inserted into the 28S subunit decoding center such that it would sterically overlap with the anticodon arm of an A-site tRNA (Fig. 3g–i), as found in case of analogous bacterial complex^27^. Minimum mitoribosomal interactions with domain IV occur in Class I complex, an intermediate number of interactions occur in Class II complex, and the maximum interactions occur in Class III complex (Fig. 3g–i). In the Class III complex, Domain IV makes contacts with multiple 12S rRNA components of the 28S subunit such as helix 24 (h24), h30, h44, and the anticodon arm of the tRNA bound in the P/P state (Fig. 3i). [We have adopted the bacterial numbering to refer to rRNA helices throughout, as they are identified by a number prefixed with an ‘h’ for the mitochondrial 12S rRNA in SSU and an ‘H’ for its 16S rRNA in LSU. The rRNA nucleotide numbering are according to Amunts and coworkers^2^.] In the 28S P-site region, aa residues S543, G544 and G545 from the loop1 region of domain IV interact with the backbone phosphates of h44 bases C1561 and G1562, while base A1078 from h24 is placed within hydrogen-bond forming distance from G544 and G545 of domain IV (Fig. 3i). The Class II and Class III maps show density for a P-site tRNA (Fig. 3e, f) bound in the classical P/P state^4,5^. However, the P-site density is relatively weak, because it represents an averaged density of the endogenously bound multi-sized tRNAs_mt_, some of which are known to have much smaller T-loops compared to their bacterial counterparts^50,51^. Nevertheless, conserved segments such as anticodon and CCA arm of tRNA could be docked into corresponding densities. Accordingly, nucleotides 33 and 34 from the anticodon of the P-site tRNA are positioned within 3 Å of residues M618 and V619 (Fig. 3i) from the loop3 region of domain IV. In the 28S head region of Class III complex, the backbone phosphates of the 12S rRNA bases U1209 and U1210 interact with aa residues S576 and N577 from the loop2 region of domain IV (Fig. 3i). The size variability in tRNAs_mt_ also affect the density corresponding to the anticodon region of the E-site tRNA_mt_ (Supplementary Notes and Supplementary Fig. 7).

Simultaneous interactions of domain IV with both the head and shoulder regions of the 28S subunit and the anticodon region of the tRNA in Complex III would stabilize the tRNA in the P site and prevent the anticodon end of translocated P-site tRNA from slipping back to the A site, as also suggested by structural studies on bacterial translocation^31,32,35,52^. The small subunit of a bacterial 70S ribosome is also found in an unrotated conformation with similar interactions in the crystallographic structure of the 70S·EF-G·GDP·FA post-translocational complex^35^, suggesting that our Class III 55S·EF-G1_mt_ complex represents an authentic post-translocation state of the human mitoribosome. The core 12S SSU rRNA regions of the mitochondrial and bacterial ribosomes that are known to interact with A- and P-site tRNAs in eubacteria are generally conserved^5,53^, and the relative orientations of bound A- and P-site tRNAs are also similar (Supplementary Fig. 8), despite the presence of a significantly altered and MRP-enriched environment around the tRNA binding sites in the LSU of the mitoribosome as elaborated under the next heading.

The tip of EF-G1_mt_ domain IV in the Class I complex is positioned ~10 Å away from 12S rRNA helices h24 and h30, closer to the 28S shoulder or the A site than its position in the Class III complex (Fig. 3g, also see Supplementary Fig. 9). Domain IV does not interact with the 28S head region in Class I complex, and the only 28S subunit element that still interacts with domain IV is the 12S rRNA h44 (Fig. 3g). This is not surprising since simultaneous interactions of domain IV with both the head and platform components would impede the head rotation, and a combination of subunit ratcheting and head swiveling help translocate the A- and P-site tRNAs into the P and E sites, respectively^31,32,47^. Unlike the Class III complex, the Class I map does not have enough density to model a P-site tRNA but superimposing the Class III P-site tRNA density onto the Class I complex suggests that the interactions of domain IV with anticodon of the P-site tRNA in the P/P state would not be established in the Class I complex (Fig. 3g). Interestingly, these contacts can be restored if the anticodon end of the P-site tRNA is moved by 6–7 Å towards the A site of the 28S subunit (Fig. 3g), which indicates that the Class I 55S·EF-G1_mt_ complex represents a key early translocation intermediate where the domain IV has moved only partially into the A site following the movement of A-site tRNA into an intermediate chimeric ap/P state^31^, preceding the Class II state (Fig. 3h) that is followed by the Class III state (Fig. 3i). Domain IV of EF-G1_mt_ synchronizes the ratcheting motion of the ribosome along with the movement of tRNAs, as it appears to closely follow the anticodon arm of the A-site tRNA during its translocation into the P site^31,32,35,52^.

### Role of P-site finger and other MRPs that directly interact with tRNAs_mt_ and EF-G1_mt_

**Fig. 4:**
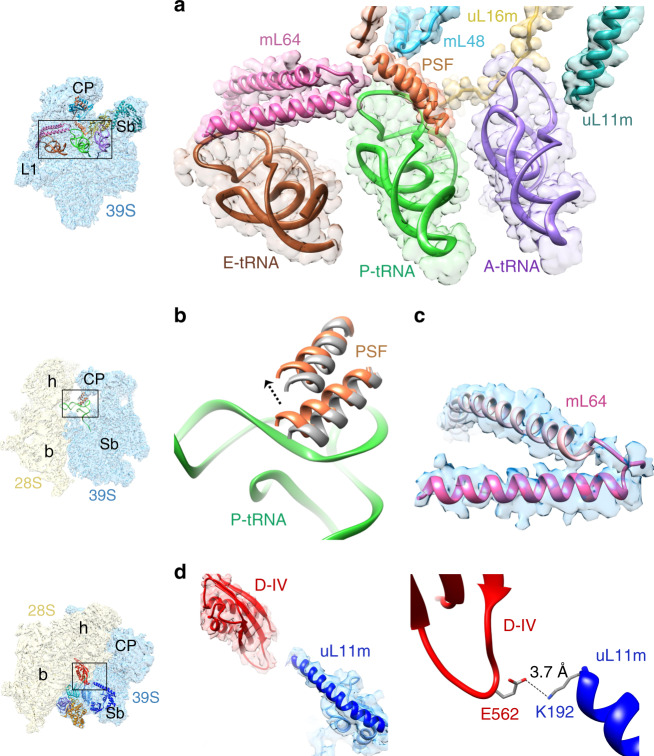
Interactions of tRNAs_mt_ and EF-G1_mt_ with mito-specific proteins and mito-specific segments in homologous proteins. **a** Interactions of the A (purple), P (green), and E (brown) tRNAs_mt_ with mito-specific proteins, PSF (orange), mL48 (blue), mL64 (pink), and mito-specific insertion/extension to homologous proteins uL11m (dark cyan) and uL16m (yellow). The A-site tRNA_mt_ position in its A/A state is derived from the 55S bovine (*Bos taurus*) map (see Supplementary Fig. 8). **b** Dynamics of PSF, which has moved by ~4 Å towards the P-site tRNA while interacting with the D and T loop regions P-site tRNA (P/P state) in the 55S·EF-G_mt_·GMPPCP complex, from its position in the vacant human mitoribosome (gray)^2^. **c** Structure of the C-terminal region (A160-E192, darker pink) of mL64 derived from Class III map (blue). **d** Interaction between the mito-specific segment of uL11m (dark blue) with the domain IV of EF-G1_mt_ (red), involving a hydrogen-bond between uL11m’s C-terminal residue K192 and E562 from domain IV. Thumbnails to the left of panels **a**, **b**, and **d** depict overall orientation of the 39S and 55S mitoribosomes. Landmarks on thumbnails: CP central protuberance, L1 uL1m stalk, Sb uL11m stalk base of the 39S LSU, h head, b body of the 28S SSU.

One of the major structural differences between the bacterial and mitochondrial ribosomes is the loss of several rRNA segments in mitochondria that are partially compensated by the acquisition of new MRPs and extensions in homologous MRPs^5,54–56^. This also changes the compositional landscape of the ribosomal intersubunit space that provides the corridor for the mRNA and tRNA movement on the mammalian mitoribosome during translation elongation. In mammalian mitochondria, protein bL5 is lost from the P site while bL25 and the A-site finger (23S rRNA helix 38) are lost from the A site^2,4,5^. The loss of these structural elements that interact with bound tRNA molecules is compensated by a unique finger-like structural element called the P-site finger (PSF) that interacts with both the A- and P-site bound tRNAs^4,5^. In the Class III complex, the PSF is found interacting with both the T-loop and the D-loop of P-site tRNA (Fig. 4a). The role of PSF appears to be to correctly position the A- and P-site tRNAs and prevent the elbow region of the P-site bound tRNA from reverting back to the A site during and after its translocation from the A to the P site. In comparison to empty 55S mitoribosomes^2,4^, the PSF has undergone a significant conformational change and moved closer towards the P-site bound tRNA in all our EF-G1_mt_-bound complexes (Fig. 4b) as well as in the mammalian mitochondrial initiation complex^9^. Tight interactions with the PSF could be one of the reasons for the frequent co-purification of mitoribosomes with a P-site bound tRNA_mt_^5^.

In our maps, we found a previously unassigned tubular density in the region between the C-terminus of mito-specific protein mL64 and the PSF^8^. This extra density is readily attributable to an α-helix-forming 32 aa residues of the C-terminus of mL64 (Fig. 4c), which extends in the 39S subunit between the P- and E-site tRNAs while interacting with the T-loop regions of both the tRNAs (Fig. 4a). Along with the mito-specific protein mL48 and the mito-specific segments of MRPs uL11m and uL16m, PSF and the C-terminus of mL64 span all three tRNA binding sites on the 39S subunit (Fig. 4a), structurally compensating for the absence of some of the bacterial homologs of r-proteins and rRNA components that are known to be involved in tRNA positioning, stabilization and translocation in the bacterial ribosome.

In all three EF-G1_mt_-bound complexes the uL11m stalk-base region within the mitoribosomal LSU moves by 5 Å towards the domain V of EF-G1_mt_ (Supplementary Fig. 4a, b), as compared to that in the empty 55S mitoribosomes^2,4^, in the initiation^9^ and in mitoribosome recycling complexes^8^. A similar movement of the uL11m region was reported for the bacterial 70S·EF-G complexes^29^. However, in the mammalian mitoribosome the conformational change is associated with a direct contact between the domain IV of EF-G1_mt_ and the mito-specific segment of uL11m (Fig. 4d). K192 from the mito-specific C-terminus α-helix of uL11m interacts with the E562 from the domain IV of EF-G1_mt_ through a hydrogen-bond interaction (Fig. 4d). Interestingly, the uniquely placed E562 is absent in EF-G2_mt_ (Supplementary Fig. 10). The presence of E562 and the mito-specific CTE in EF-G1_mt_ and their absence in EF-G2_mt_, along with presence of four small insertion segments within corresponding domains II and III of EF-G2_mt_ (Supplementary Fig. 10), appear to be the key differences that confer specificity to these two factors for their roles in elongation and recycling steps, respectively.

### Role of the C-terminal extension in EF-G1_mt_

**Fig. 5:**
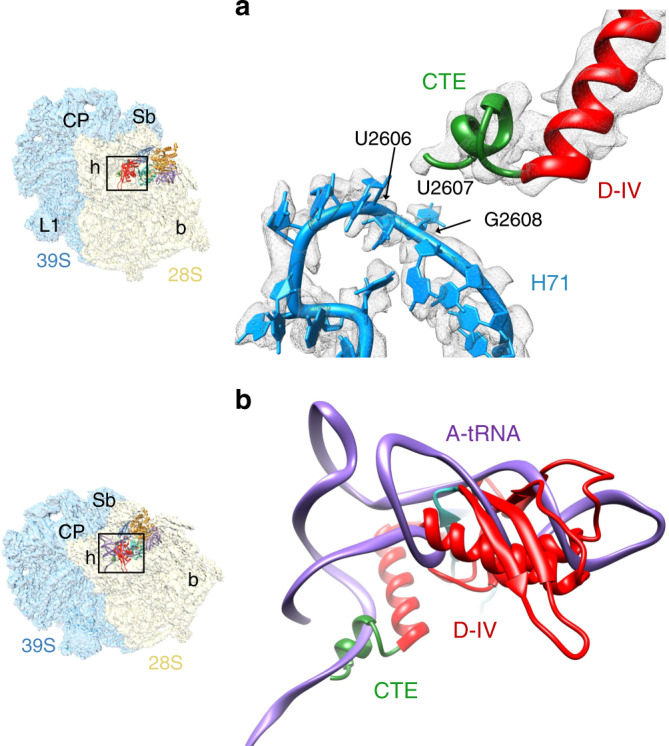
Interaction of mito-specific C-terminal extension of EF-G1_mt_ with rRNA and tRNA. **a** The C-terminal extension (CTE, green) of domain IV (red) interacts with the 16S LSU rRNA helix 71 (H71, blue). **b** Superposition of the A-site bound tRNA_mt_ (purple) onto the EF-G1_mt_-bound human 55S structure revealed that while bulk of domain IV of EF-G1_mt_ overlaps with the anticodon-stem-loop region, the CTE partially overlaps with the CCA arm of the A-site tRNA_mt_. Landmarks on thumbnails to the left, which depict the overall orientations of the 55S mitoribosome, are as in Fig. 4.

Both the Class I and Class III complexes show an additional density adjacent to the conserved C-terminal end of the EF-G1_mt_ domain IV that could readily accommodate its mito-specific C-terminal extension (CTE) (Fig. 5a), which is not resolved in the Class II complex. The lysine-rich CTE folds into an α-helix and extends into the 39S subunit enabling the EF-G1_mt_ to interact with rRNA and tRNA_mt_ segments that would be inaccessible to the bacterial EF-Gs. The CTE is positioned close to the nucleotides U2606-G2608 segment of the 16S LSU rRNA helix 71 (H71) (Fig. 5a). In its current orientation, the CTE would overlap with the inner bend of A-site tRNA_mt_ elbow primarily involving tRNA_mt_’s CCA arm (Fig. 5b), suggesting that the CTE plays a direct role in the movement of the CCA arm of the A-site bound tRNA_mt_. The interaction of lysine-rich CTE with H71 would also prevent the reverse translocation of the P-site tRNA to the A site. The fact that EF-G1_mt_ remains active on the *E. coli* ribosomes, but *E. coli* EF-G remains inactive on mitoribosomes^57^, suggests that the observed interaction of EF-G1_mt_’s CTE with the mitoribosome and the A-site tRNA_mt_ in our structure could also be associated with EF-G1_mt_’s GTPase activity on the ribosome. A significantly altered landscape of the mitoribosomal intersubunit space described in the previous section and the location of EF-G1_mt_’s CTE on the mitoribosome suggest that the MRPs and translational factors have coevolved with its unique tRNAs${}_{{\mathrm{mt}}}$ to structurally and functionally compensate for the lost bacterial RNA segments.

### Conformational changes at the nascent polypeptide-exit site

The newly synthesized protein chain exits the ribosome through a tunnel-like feature in the large subunit^5,58,59^ known as the nascent polypeptide-exit tunnel (NPET). The NPET originates from the peptidyltransferase center (PTC) and ends on the opposite side at the solvent interface, which is referred to as the polypeptide-exit site (PES). The structural composition of this tunnel is substantially different between the bacterial and the mammalian mitochondrial ribosomes^2,4,5^. Domains I and III of the 23S rRNA that line the bottom portions of NPET in bacteria are greatly reduced in the analogous mitochondrial 16S LSU rRNA^5,53^. The loss of these important structural components surrounding the tunnel is compensated through the acquisition of larger bacterial r-protein homologs with extended N and C termini^5,54,56^. A mito-specific protein mL45 is also present near the PES^2,4^. During the initiation phase, the entire NPET is blocked by the insertion of the N-terminus (NT) residues 38–64 of mL45 into the NPET^9^. The N-terminal region of mL45 also interacts with MRPs uL23m and uL24m near PES. Mutational studies have shown that deletion of the mL45 NT severely inhibits mitochondrial translation^9^.

**Fig. 6:**
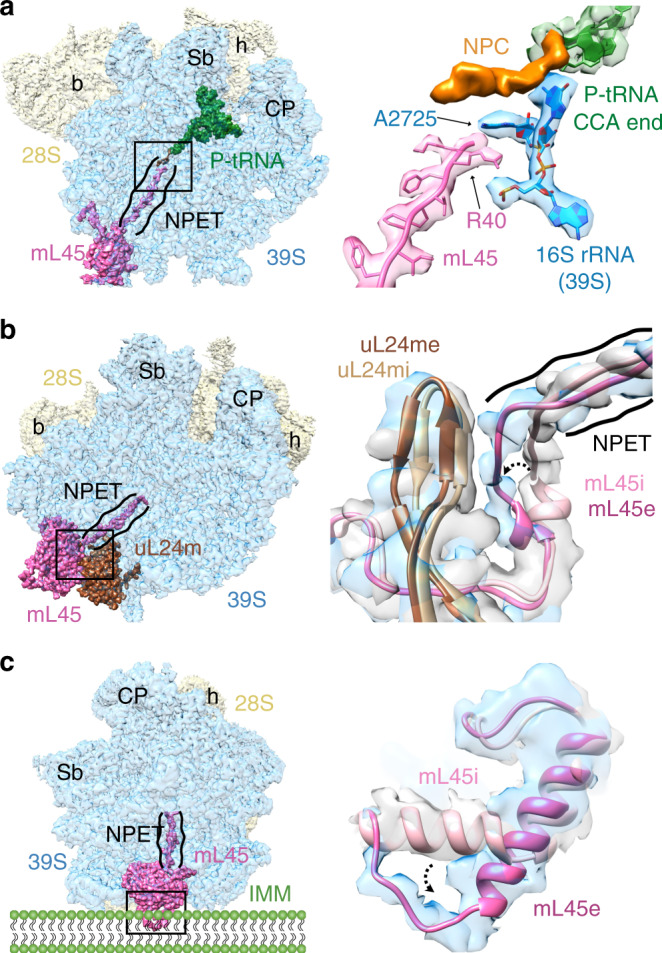
Location of the nascent polypeptide chain and conformational changes in mL45 between the initiation and elongation complexes. **a** A density linked to the CCA end of the P-site tRNA (green) was identified as NPC (orange) that approaches the N-terminus (NT) of ribosomal protein mL45 (pink). A2725 from the 16S LSU rRNA (blue) is positioned between the NT of mL45 and the nascent polypeptide chain (NPC). **b** The N terminus region (R61-D73) of mL45 in the elongation complex (mL45e, deep pink) has shifted substantially toward uL24m, as compared to its position in the initiation complex (mL45i, light pink)^9^. The shift in mL45 is accompanied by a small movement in uL24m between the initiation (uL24i, light brown) and elongation (uL24e, dark brown) states. **c** Conformational change in the core region of mL45. Curved arrows in panels **b** and **c** point to observed movements from initiation to elongation complexes. Thumbnails in panel **a**, **b**, and **c** depict the overall orientation of the 55S mitoribosome where the 28S SSU (semitransparent yellow) is placed behind the 39S LSU (semitransparent blue), with relevant MRPs highlighted. Landmarks on the thumbnails are as in Fig. 4. Depiction of inner mitochondrial membrane (IMM) in panel **c** provides context to the location of the observed conformational change.

Though the blocked NPET might not pose any problem for an initiating mitoribosome, a vacant tunnel would be necessary to accommodate the growing nascent polypeptide chain during the translation elongation phase. In all our complexes, we found an unassigned density that is connected to the CCA end of the P-site tRNA and reaches close to the NT of the ribosomal protein mL45 inside the NPET (Fig. 6a). This density that could accommodate up to 5 aa residues can be readily attributed to a nascent peptide chain (NPC). In our structure, a conserved adenine residue (A2725) from a loop region between the 16S LSU rRNA helices H73 and H74 intercalates between the NT of NPC and aa R40 from the NT of mL45 (Fig. 6a). By simultaneously interacting with the NPC and NT of mL45, A2725 might play a crucial role in triggering a conformational change in the large mitoribosomal subunit that eventually results in the retraction of NT of mL45 from the NPET to make room for the growing NPC. Indeed, we observed a significant conformational change involving aa residues R61 to D73 (Fig. 6b). Compared to their position in the initiation complex^9^, these residues have moved substantially, ~9 Å away from the tunnel and toward uL24m, which also shifts in conjunction with the mL45 movement. The EF-G1_mt_-induced conformational change in the large subunit captured in our structure likely represents a functional state, as mL45 prepares to retrieve its NT from the NPET to allow the insertion of incoming nascent polypeptide from the PTC side of the NPET.

In addition, the residues T101-Y128 located in the core region of mL45 undergo a large conformational change (Fig. 6c) between the initiation^9^ and our elongation complexes. In the initiation complex, these residues form two separate α-helices with an angle of ~120° between them. Of these, residues T109–T115 from the N-terminal helix rotate by ~60° to become part of a single long α-helix in the elongation complex, leaving residues S101-R108 in an open conformation. This large conformational change, involving a secondary structure rearrangement, may be necessary for anchoring of the mammalian mitoribosome to the inner mitochondrial membrane (IMM). mL45 happens to be the homolog of Mba1, the IMM-associated receptor necessary for the co-translational insertion of nascent polypeptides into the IMM in yeast^60^. Interestingly, this positively charged segment (aa residues 101–114) of mL45 has been implicated in mediating the association of 55S mitoribosomes with the negatively charged lipid content of the IMM through charge-based interactions^61^, which could be accompanied by the observed conformational change in this study. Furthermore, the C-terminal region of the uL23m that interacts with the mL45 NT has undergone a large conformational change where its α-helix involving aa residues A123-R137 has moved by ~20 Å toward the mL45 NTE. It appears that this α-helix movement is necessary to clear the path for the displacement of mL45’s NTE from the exit tunnel (Supplementary Fig. 11a). However, a comparison of the sequences of uL23m in human with other mammals (Supplementary Fig. 11b) suggests that this major conformational difference could be species-specific.

In summary, our study presents the most complete structure for the human 55S mitoribosome, and shows that the EF-G1_mt_-bound mitoribosome can adopt at least three different conformations irrespective of the GTP hydrolysis state. The major variation occurs in the relative orientation of its entire 28S subunit, or only its head domain, suggesting an unusual adaptability of the 28S subunit during translocation (Fig. 3). Direct structural evidence is presented that the mito-specific components in both the mitoribosome and EF-G1_mt_ are involved in tRNA_mt_ translocation. Our study also shows how mito-specific ribosomal proteins, such as PSF and mL64 in the mitoribosome’s tRNA_mt_ interaction sites (Fig. 4), and the addition of a mere 11 aa residues in the C-terminus of EF-G1_mt_ (Fig. 5), allow the mitochondrial translation system to adapt to a massive reduction in mitoribosomal RNA components as compared to their bacterial counterparts. For example, the absence of 23S rRNA helix 38, also known as the A-site finger that dynamically interacts with both A- and P-site tRNAs during the tRNA translocation in eubacteria, is structurally and functionally compensated by the PSF protein in the mammalian mitoribosome. Similarly, the missing eubacterial E-site tRNA interacting rRNA components^53^ are replaced by protein mL64. Finally, the large conformational changes between the initiation and elongation states involving mito-specific protein mL45 in the NPET’s exit site (Fig. 6), seem to be associated with the mitoribosomal anchoring to the IMM.

## Methods

### Isolation of mitochondria from HEK cells

Mitochondria were isolated from human embryonic kidney cells lacking *N*-acetyl-glucosaminyltransferase I (HEK293S GnTI)^8^ that were cultured in roller bottles using FreeStyle^TM^293 media (Gibco, Life Technologies) supplemented with 5% fetal bovine serum (Gibco, Life Technologies). After centrifugation at 1000 × *g* for 7 min, the HEK293S GnTI cell-pellet was transferred to a glass homogenizer and resuspended in buffer containing 50 mM HEPES-KOH pH 7.5, 10 mM KCl, 1.5 mM MgOAc, 70 mM sucrose, 210 mM mannitol, 1 mM EDTA, 1 mM EGTA, 1 mM DTT, and 1 mM PMSF. After homogenization, the supernatant was separated from the cell debris by spinning at 950 × *g* for 15 min. The supernatant was then spun at 11,000 × *g* for 15 min, and the resulting pellet that contains crude mitochondria was resuspended in SEM buffer (250 mM sucrose, 20 mM HEPES-KOH pH 7.5, 1 mM EDTA, and 1 mM EGTA). DNase I (3 units/ml) was added to the crude mitochondria and incubated at 4 °C for 1 h. A discontinuous gradient was prepared in a Beckman polyallomer tube by layering 2.5 ml of 60%, 4 ml of 32%, 1 ml of 23%, and 1 ml of 15% sucrose solutions in buffer containing 10 mM HEPES-KOH pH 7.5 and 1 mM EDTA. DNase-treated sample was loaded on the discontinuous gradient and centrifuged for 1 h at 135,000 × *g* using Ti70 rotor in a Beckman ultracentrifuge. The brownish-orange layer containing pure mitochondria was carefully separated and re-suspended in SEM buffer.

### Isolation of mitoribosomes from mitochondria

Mitoribosomes were isolated^8^ by adding four volumes of lysis buffer (25 mM HEPES-KOH pH 7.5, 100 mM KCl, 25 mM MgOAc, 1.7% Triton X-100, 2 mM DTT and 1 mM PMSF) to the mitochondrial-pellet and then incubating for 15 min at 4 °C. The sample was centrifuged at 30,000 × *g* for 20 min and the supernatant was loaded on top of 1 M sucrose cushion in buffer (20 mM HEPES-KOH pH 7.5, 100 mM KCl, 20 mM MgOAc, 1% Triton X-100, and 2 mM DTT). After centrifugation for 17 h at 90,000 × *g* using Ti70 rotor in Beckman ultracentrifuge, a minimal volume of Mitobuffer (20 mM HEPES-KOH pH 7.5, 100 mM KCl, 20 mM MgOAc, and 2 mM DTT) enough to dissolve the pellet was added. 10–30% continuous sucrose density gradients were prepared in Mitobuffer, using the gradient making apparatus (C.B.S. Scientific Co.). The resuspended pellet was subjected to 10–30% continuous sucrose density gradient centrifugation at 60,000 × *g* for 17 h using Sw32 rotor in Beckman ultracentrifuge. The gradient was fractionated on ISCO gradient analyzer (Teledyne ISCO, Inc), and the fractions corresponding to the mitoribosomes were collected and pooled. Finally, the pooled mitoribosomes were concentrated by spinning them at 130,000 × *g* for 6 h using Ti70 rotor, and the pellet was resuspended in Polymix buffer (5 mM HEPES-KOH pH 7.5, 100 mM KCl, 20 mM MgOAc, 5 mM NH_4_Cl, 0.5 mM CaCl_2_, 1 mM DTT, 1 mM spermidine, and 8 mM putrescine)^62^.

### Cloning and expression of human EF-G1_mt_

An expressed sequence tag coding for human EF-G1_mt_ was obtained from GeneCopoeia (No. GC-W1058). Using PCR, the sequence corresponding to the mature form of EF-G1_mt_ (amino acids 36–751) was amplified by employing forward 5′-GGAATTCCATATGTCTTCATCAGGGGTGATTCC-3′ and reverse 5′-AACCGCTCGAGTTCTTGGCTTTTCCTTTTTTAAC-3′ primers^39^. The PCR product was cloned into pET 21c (+) (Novagen) and this vector provides a sequence encoding six His residues (His-tag) at the C-terminus. The resulting construct was transformed into *E. coli* ER2267 and subsequently transformed into *E. coli* BL21(DE3) (RIL) for over-expression.

### Purification of human EF-G1_mt_

The cultures were grown to mid-log phase and induced with 50 μM isopropyl-1-thio-d-galactopyranoside (IPTG). After centrifugation at 5,000 rpm for 15 min at 4 °C, the cells were harvested, shock-frozen, and stored at −80 °C. The frozen cells were disrupted by grinding with double the cell weight of Alumina Type A-5 (Sigma) for a total of 20 min. The paste was resuspended in Buffer B (50 mM Tris–HCl, pH 7.6, 40 mM KCl, 7 mM MgCl_2_, 7 mM β-mercaptoethanol, 0.1 mM phenylmethylsulfonyl fluoride, and 10% glycerol), and the debris was removed by centrifugation at 10,000 rpm at 4 °C for 10 min. This is followed by DNase I (5 μg/mL) treatment and centrifugation at 15,000 rpm at 4 °C for 20 min. The resulting supernatant was mixed with 0.6 mL of a 50% slurry of Ni–NTA resin equilibrated in Buffer B and relatively pure EF-G1_mt_ was obtained using affinity chromatography^39^. In order to achieve high-level purity, ion exchange chromatography technique was employed where the partially purified EF-G1mt from the Ni–NTA purification was processed on a cation exchange TSKgel SP-5PW column (TosoHaas, Japan).

### Preparation of the human mitoribosome•EF-G1_mt_•GMPPCP complex

To obtain the 55S·EF-G1_mt_ complex, a non-hydrolysable analog of GTP, GMPPCP, was used to lock EF-G1_mt_ on the mitoribosomes. The complex was formed by incubating 150 nM 55S mitoribosomes with 5 μM EF-G1_mt_ and 1 mM GMPPCP (Sigma-Aldrich, USA) at 37 °C for 5 min in the HEPES polymix buffer.

### Cryo-electron microscopy and image processing

In all, 4 μl of the 55S·EF-G1_mt_•GMPPCP complex was applied to Quantifoil holey copper 1.2/1.3 grids that were pre-coated with a thin layer (~50 Å thick) of home-made continuous carbon film and glow-discharged for 30 s on a plasma sterilizer. After incubating the grids for 15 s at 4 °C and 100% humidity, they were blotted for 4 s and immediately flash-frozen into the liquid ethane with the help of a Vitrobot (FEI company). Data were collected on a Titan Krios electron microscope (FEI company) equipped with a Gatan K2 summit direct-electron detecting camera at 300 kV. We used a defocus range of −1.0 to −3.0 µm at a calibrated magnification of ×105,000, yielding a pixel size of 1.0961 Å. A dose rate of 7 electrons per pixel per s and an exposure time of 10 s resulted in a total dose of 69.2 e/Å^2^. All the downstream processing of data was done using CryoSPARC^63^. Full-frame motion correction was applied to all 50 movie frames corresponding to each of the 6,671 micrographs. After determining their contrast transfer function (CTF) using CTFFIND4^64^, bad images were deselected. From the remaining 6649 micrographs, 1,611,0847 particles were picked using the auto-pick function, and after local motion correction, 1,262,274 particles remained. This step was followed by the reference-free 2D classification and finally 851,131 good particles were retained based on the 2D averages. The initial 3D reconstruction and refinement yielded 2.74 Å resolution 55S map (Supplementary Fig. 1), with its 39S subunit showing an overall resolution of 2.68 Å (Supplementary Fig. 2), and the local resolution in the core regions of the 39S subunit were found to be in the 2.5–2.6 Å range (Supplementary Fig. 2). However, the 28S subunit was relatively poorly resolved. Reference-based 3D classification was employed to separate 55S mitoribosomes (289,982 particles) from 39S subunits (408,686 particles) and 28S subunits (152,463 particles). Particles corresponding to the 55S mitoribosomes were further subjected to multiple rounds 3D classification that yielded three stable classes, Class I (99,804 particles), Class II (25,755 particles), and Class III (150,347 particles), and allowed us to remove a small population of 14,076 bad particles. After 3D refinement Class I, Class II and Class III yielded a final resolution of 2.97 Å, 3.96 Å, and 2.96 Å, respectively, and all of the three classes showed strong densities that could be readily attributed to a bound EF-G1_mt_. After masked local refinements, the 28S and 39S subunits from the Class I complex were refined to 3.15 Å and 2.91 Å, respectively; the 28S and 39S subunits from the Class II complex were refined to 4.82 Å and 3.77 Å, respectively; and the 28S and 39S subunits from the Class III complex were refined to 3.04 Å and 2.87 Å, respectively (Supplementary Fig. 2). The Gold-standard criterion of 0.143 FSC cutoff^65^ was used to report all resolutions.

### Model building and optimization

Coordinates corresponding to the small and large subunits of the published human mitoribosome (PDB ID: 3J9M)^2^ were used as the initial template. The higher resolution of our maps (Supplementary Fig. 3) enabled us to build multiple protein and rRNA segments that were not present in the previous human mitoribosome structures^2,8,66^. Highly resolved secondary structural elements (SSE) and amino acid side-chain features guided the manual building of the majority of protein models using UCSF Chimera 1.14^67^ and COOT^68^. For modeling the relatively low-resolved regions such as the L7/L12 stalk proteins and the C terminal domain (CTD) of L12 from the large subunit and protein mS39 from the small subunit, homologous structures from the porcine mitoribosome^9^ were used as a template. Additional segments (75 rRNA nts and 1082 aa residues) that were absent in the previous human mitoribosome structures were modeled de novo. These new rRNA segments were built in ModeRNA server^69^, using corresponding segments wherever available from the porcine mitoribosome^9^ as a template. For building the P- and E-site tRNAs, the high-resolution structure of yeast tRNA^Phe^ (PDB ID: 1EhZ)^70^ was used as template to generate tRNA_mt_^Phe^. The tRNA_mt_^Phe^ was docked manually and rigid body fitted into the corresponding cryo-EM density using Chimera 1.14^67^. Owing to sub-optimal occupancies and inherent heterogeneity within the endogenously bound tRNAs_mt_, the resolution corresponding to the tRNA_mt_ densities were relatively low to allow any sequence-specific modeling  of the P- and E-site tRNAs_mt_. Homology models of EF-G1_mt_ generated in I-TASSER^71^ were used as the initial template. Regions in the homology model that do not fully accommodate into the corresponding EF-G1_mt_ density were modeled de novo using Chimera 1.14^67^ and COOT^68^. Lower resolution in our cryo-EM maps corresponding to the density of EF-G1_mt_ C terminal extension (CTE) restricted modeling of this region at the side-chain level but permitted building the carbon backbone guided by recognizable SSEs. For the final optimization of the models into the cryo-EM densities, we used the “Real-space refinement” function in PHENIX^72^. The models were validated using Molprobity server^73^, and the overall statistics of EM reconstruction and molecular modeling are listed in Supplementary Table 1.

### Reporting summary

Further information on research design is available in the Nature Research Reporting Summary linked to this article.

## Supplementary information

Supplementary Information

Peer Review

Reporting Summary

## Data Availability

The data that support this study are available from the corresponding authors upon reasonable request. The cryo-EM maps and atomic coordinates have been deposited in the Electron Microscopy and PDB Data Bank (wwPDB.org) under accession codes EMD-21233 [https://www.ebi.ac.uk/pdbe/entry/emdb/EMD-21233] and PDB 6VLZ [10.2210/pdb6VLZ/pdb] for the EF-G1_mt_-bound 55S mitoribosome (Complex I), and EMD-21242 [https://www.ebi.ac.uk/pdbe/entry/emdb/EMD-21242] and PDB 6VMI [10.2210/pdb6VMI/pdb] for the EF-G1_mt_-bound 55S mitoribosome (Complex III). Cryo-EM maps of the Complex II and bovine 55S mitoribosome have been deposited with accession codes EMD-22212 [https://www.ebi.ac.uk/pdbe/entry/emdb/EMD-22212] and EMD-22209 [https://www.ebi.ac.uk/pdbe/entry/emdb/EMD-22209], respectively. All raw micrographs and particle images used in 3D reconstructions will be made available through empiar, an electron microscopy public image archive, https://www.ebi.ac.uk/pdbe/emdb/empiar/.
