# Supplementary material for: Structures of the human mitochondrial ribosome bound to EF-G1 reveal distinct features of mitochondrial translation elongation
Source: Nat Commun. 2020 Jul 31;11:3830. doi: 10.1038/s41467-020-17715-2 (PMC7395135; doi:10.1038/s41467-020-17715-2)
Supplement: Supplementary file 1 — Supplementary Information [file 41467_2020_17715_MOESM1_ESM.pdf]

Supplementary Information

Structures of the human mitochondrial ribosome bound to EF-G1 reveal distinct features of mitochondrial translation elongation

Ravi Kiran Koripella[1,#], Manjuli R. Sharma[1,#], Kalpana Bhargava[2], Partha P. Datta[1], Prem S. Kaushal[1], Pooja Keshavan[1], Linda L. Spremulli[2], Nilesh K. Banavali[1,3], and Rajendra K. Agrawal[1,3,*]

[1]Wadsworth Center, New York State Department of Health, Empire State Plaza, Albany, New York 12201;

[2]Department of Chemistry, Campus Box 3290, University of North Carolina, Chapel Hill, NC; and

[3]Department of Biomedical Sciences, University at Albany, SUNY, New York 12201-0509.

[#]Authors contributed equally

*Corresponding author: rajendra.agrawal@health.ny.gov

Supplementary Methods

Cryo-electron microscopy and image processing for the control human 55S mitoribosome: The same batch of human mitoribosome that was used for preparation of the 55S•EF-G1$_{mt}$•GMPPCP complex was used for the determination of cryo-EM structure of the control (EF-G1$_{mt}$ unbound) human 55S mitoribosome. The grid preparation and image processing steps for the control (EF-G1$_{mt}$ unbound) human 55S mitoribosome were the same as for the 55S mitoribosome•EF-G1$_{mt}$•GMPPCP complex, except that the cryo-EM data was collected at 300 kV on a JEOL 3200 FSC electron microscope, using a Gatan K2 summit direct-electron detecting camera. A dose rate of 3.5 electrons per pixel per s and an exposure time of 4 s resulted in a total dose of 9 eÅ^{-2}. A defocus range of -1.0 to -3.0 µm was used at a magnification of 25,000 X with a pixel size of 1.25 Å. Built-in full-frame motion correction within the SerialEM software [1] was applied to 40 movie frames to obtain each aligned micrograph. After visual inspection and the CTF fit using CTFFIND4[2], 2,436 micrographs were selected, from which 505,387 particles were auto-picked and classified using reference-free 2D classification using cryoSPARC [3], and 147,221 particles were retained based on the 2D averages. The initial 3D reconstruction and refinement yielded 7.4 Å resolution 55S map with a weaker density for the 28S SSU region (Supplementary Figure 6). Reference-based 3D classification was employed to separate 55S mitoribosomes (66,356 particles) from 39S LSU (45,100 particles) and 28S SSU (35,765 particles). Refinement of the 55S mitoribosome resulted in an 8.3 Å resolution map. 3D classification of the 55S mitoribosome particles resulted in two major classes, a smaller class with 28,896 particles that refined to 10.4 Å resolution showed an un-rotated 28S SSU head, similar to class III of the 55S•EF-G1$_{mt}$•GMPPCP complex, and a slightly larger class with 37,460 particles that refined to 8.6 Å showed a partially ratcheted 28S SSU (without head swiveling), as compared to class I of the 55S•EF-G1$_{mt}$•GMPPCP complex. Both classes showed density for the E-site tRNA, but the class with unrotated SSU also showed stronger density for the P-site tRNA. The conformation of one of our two control classes match with previously reported classes I of Amunts and coworkers[4].

Preparation and purification of the bovine mitoribosome: Bovine (*Bos taurus*) mitoribosomes were prepared following the previously published protocol as described below.[5]

The mitochondria were isolated from bovine liver by homogenization, followed by differential centrifugation in buffer containing 4 mM Hepes- KOH (pH 7.6), 440 mM mannitol, 2 mM EDTA, and 140 mM sucrose. Then the mitochondrial pellets were homogenized in buffer containing 0.26 M sucrose, 40 mM KCl, 15 mM MgCl2, 15 mM Tris·HCl (pH 7.6), 6 mM β-mercaptoethanol, 0.8 mM EDTA, 0.05 mM spermine, and 0.05 mM spermidine, and were centrifuged at 15, 000 rpm in a Sorvall SS34 rotor for 45 min. The supernatant was layered on a 34 % sucrose cushion after the concentration of KCl in the supernatant was increased to 300 mM and centrifuged at 35,000 rpm for 17 h in a Beckman Type Ti 70 rotor. Pellets were dissolved in buffer containing 20 mM Hepes- KOH (pH 7.6), 100 mM KCl, 20 mM MgCl2, and 2 mM DTT and crude ribosomes were subjected to 10–30% sucrose gradient centrifugation at 22,000 rpm using a Beckman SW32 rotor for 16 h. The 55S bovine mitoribosome fractions were collected after fractionating the sucrose gradient in Teledyne ISCO gradient analyzer. The pooled fractions were pelleted by ultracentrifugation at 42,000 rpm for 6 h in a Beckman type Ti70 rotor. The 55S ribosomes were stored at −80 °C in storage buffer containing 20 mM Hepes-KOH (pH 7.6), 20 mM MgCl2, 40 mM KCl, 20 mM DTT, and 5% glycerol.

Cryo-electron microscopy and image processing for the bovine 55S mitoribosome:
The cryo-EM grids were prepared by applying 4 μL of the bovine 55S mitoribosome suspension (32 nM) onto the Quantifoil Holey carbon copper grid using Vitrobot (FEI). Data were collected on a 300-kV POLARA field emission gun electron microscope equipped with Gatan K2 direct electron detector camera at calibrated magnification of 31,558. corresponding to 1.66 Å pixel size. A total 2,901 micrographs were collected at a dose rate of 50 electrons per Å^2 per second. Movie frames were aligned using whole-image motion correction.[6] and image processing was performed using RELION.[7] The CTFFIND3 was used to calculate the CTF correction parameters.[2] 1,789 micrographs were selected after manual inspection for astigmatism, contamination and poor contrast. 208,047 particles were picked using semi-automated particle picking implemented in RELION[7], after 2D classification and initial 3D classification, 61,640 best particles were used for 3D refinement gave 6.1 Å resolution map (Supplementary Figure 8). The 61,640 particles were further classified into four classes. While class 1 showed densities for both A- and P-site tRNAs, Classs 2 and 3 looked very similar, as both contained fragmented densities for E-site tRNA in addition to A and P-site tRNAs. Therefore, classes 2 and 3 were

merged and refined to 6.8 Å resolution according to Fourier shell correlation (FSC) 0.143 cutoff criterion.[8] The bovine 55S maps were used only for comparative evaluation of tRNA distribution in different binding sites, and for deriving the A-site tRNA position.

Supplementary Notes

Conformational state of the $tRNA_{mt}$ bound in the E site:

We found a partial density corresponding to a bound E-site $tRNA_{mt}$ in all three maps. This E-site $tRNA_{mt}$ density is better resolved at the elbow and the CCA regions but poorly resolved in the elbow and anticodon stem-loop (ASL) regions (Figure 3). This is in sharp contrasts to the situation in cytoplasmic ribosomes, where strong densities are consistently observed for the entire E-site tRNA[9-12], suggesting that the deacylated-$tRNA_{mt}$ while leaving the P site during the EF-$G1_{mt}$-dependent translocation stays only transiently at the putative E site, which has low affinity for the incoming deacylated $tRNAs_{mt}$. The reduced affinity for E-site $tRNA_{mt}$ is supported by the observation that 11 of the 12 interacting sites for the deacylated-tRNA at the E site of the large subunit rRNA in eubacteria are absent in the mammalian mitoribosome.[13] However, one of the rRNA nucleotides, C2394 in the eubacterial 23S rRNA, is universally conserved and is known to be essential for the tRNA binding at the E site.[14] The CCA arm of the E-site $tRNA_{mt}$ is held tightly in position through strong interactions from bases C2899 (C2394 in *E. coli*), C2900, A2901, G2909 and A2910 from the 16S rRNA H88 helix (Supplementary Figure 7). Similar interactions are observed in the bacterial E site.[10,15,16] The possible explanation for the relatively poor resolution of the ASL region of the $tRNAs_{mt}$ is the dramatic variations in size of the $tRNAs_{mt}$.[17] When the smaller sized $tRNAs_{mt}$ are held tightly in place by simultaneously interacting with universally conserved nucleotides of H88 through their CCA end and with MRP uL1m through their elbow regions, it is likely that the contacts between the ASL of the smaller $tRNAs_{mt}$ and the mRNA are more readily disrupted than that in a typical full size $tRNA_{mt}$ (Supplementary Figure 7). Such a size variation would result in reduced average density for the ASL region of the E-site $tRNAs_{mt}$, as observed in our maps.

A high degree of head swiveling is known to be essential for tRNA translocation from the P site to the E site since it widens a gate-like constriction formed between the 16S rRNA bases A790 (h24) from the platform region and G1338-U1341 (h29) from the head region of the bacterial 30S

subunit, which otherwise would sterically block the movement of the anticodon stem-loop from the P to the E site.[12,18,19] Except for the A1340, which has been replaced by a Uracil (U1424), all other interacting residues are conserved in the 12S rRNA of the 28S subunit in the human mitoribosome. Interestingly, the h29 of 12S rRNA is slightly shorter than that in the eubacterial 16S rRNA, but this change in overall dimension of h29 does not impact the gating mechanism between the P and E sites, as an analysis of the 28S subunits in our structures, and 28S subunit from different mammalian mitoribosomal structures[4,20-24] revealed that the size of constriction between h24 and h29 varies between 17 Å and 26 Å among these mitoribosome structures, in line with the observations made for the 30S subunit of the bacterial ribosomes.[12,18,19]

Conformational changes in the L11-stalk-base region:

The movement of uL11m region towards domain V is one of the major conformational changes in the large ribosomal subunit induced by the binding of EF-G1$_{mt}$ (Supplementary Figure 4a). By positioning adjacent to the uL11m stalk-base region, domain V of EF-G1$_{mt}$ forms extensive interactions with several 39S subunit elements, and thereby provides major anchoring points for the stable binding of EF-G1$_{mt}$ on the 55S mitoribosome. Domain V interacting partners from the large subunit include the uL11m N-terminal domain (NTD) and the 16S rRNA helices H43, H44, H89 and H95 (Supplementary Figure 4b).

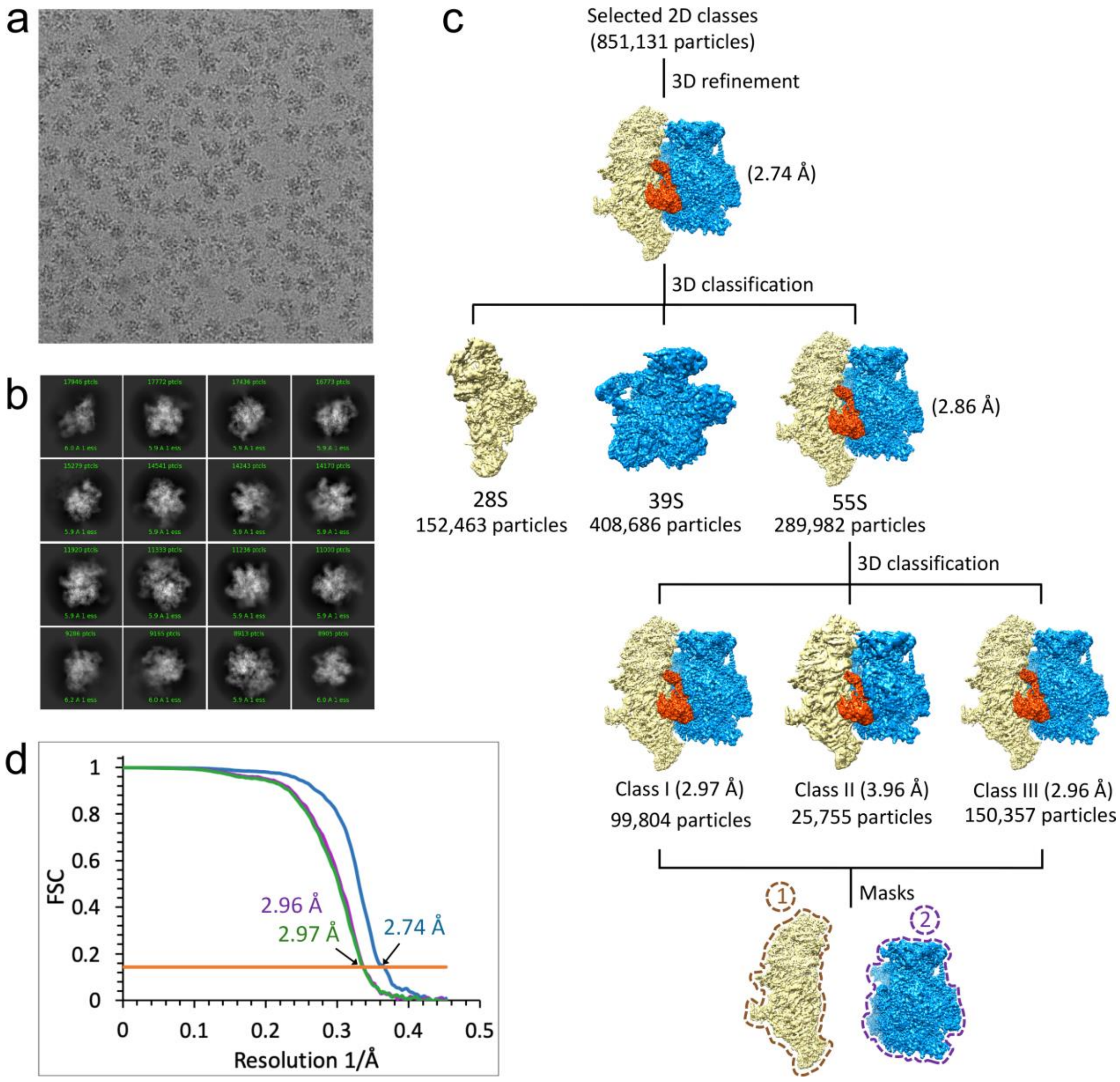

Supplementary Figure 1. Processing of the 55S•EF-G1$_{mt}$•GMPPCP complex cryo-EM dataset. (**a**) A representative electron micrograph showing human mitoribosomal 55S•EF-G1$_{mt}$•GMPPCP complexes. (**b**) Representative two-dimensional (2D) class averages used in three-dimensional (3D) reconstructions. (**c**) A flow-chart of cryo-EM maps generated during 3D classifications and refinements. The selected 2D averages (851,131 particles) were subjected to an initial 3D refinement that yielded a 2.74 Å resolution 55S mitoribosomal map. Using reference-based 3D classification, the intact 55S mitoribosomes (289,982 particles) were separated from the 39S subunits (408,686 particles) and the 28S subunits (152,463 particles). Further classification of the 55S mitoribosomes yielded three stable classes with distinct

conformational states that were refined to 2.97 Å (Class I), 3.96 Å (Class II), and 2.96 Å (Class III). Maps corresponding to Classes I and III were used for model building, after further improving resolution of individual subunits by applying local refinement using masks (see Supplementary Figure 2c-e). Mask 1 (brown) was focused on the 28S subunit (yellow) and mask 2 (purple) on the 39S subunit (blue). (**d**) Fourier-shell correlation (FSC) plots of the combined (blue), Class I (green) and Class III (purple) maps.

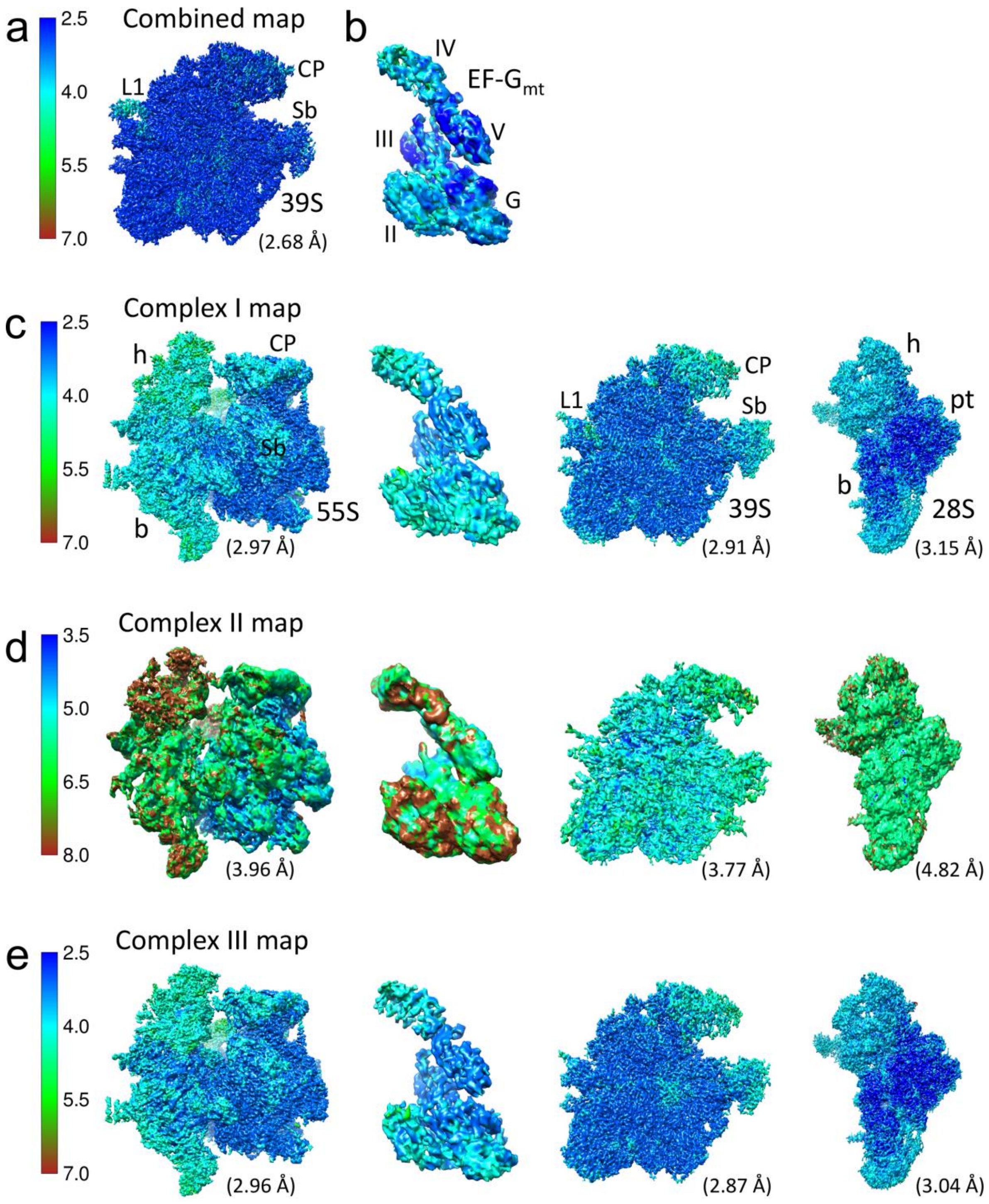

Supplementary Figure 2. Local surface resolution of the 55S•EF-G1$_{mt}$•GMPPCP complexes. (**a**) 39S subunit derived from the whole 55S data set (289,982 particles), after local 3D refinement using mask. (**b**) EF-G1$_{mt}$ derived from the same combined dataset. (**c** - **e**) Panels **c** to **e** show local resolution maps for the three 55S•EF-G1$_{mt}$•GMPPCP complexes. In each of the

c-e panels, in addition to the full 55S•EF-G1$_{mt}$•GMPPCP complex (left side), shown are its individually masked and refined components in the following order (left to right): EF-G1$_{mt}$, 39S LSU and 28S SSU. The overall FSC resolution values for the maps of the 55S and its two subunits are indicated in brackets.

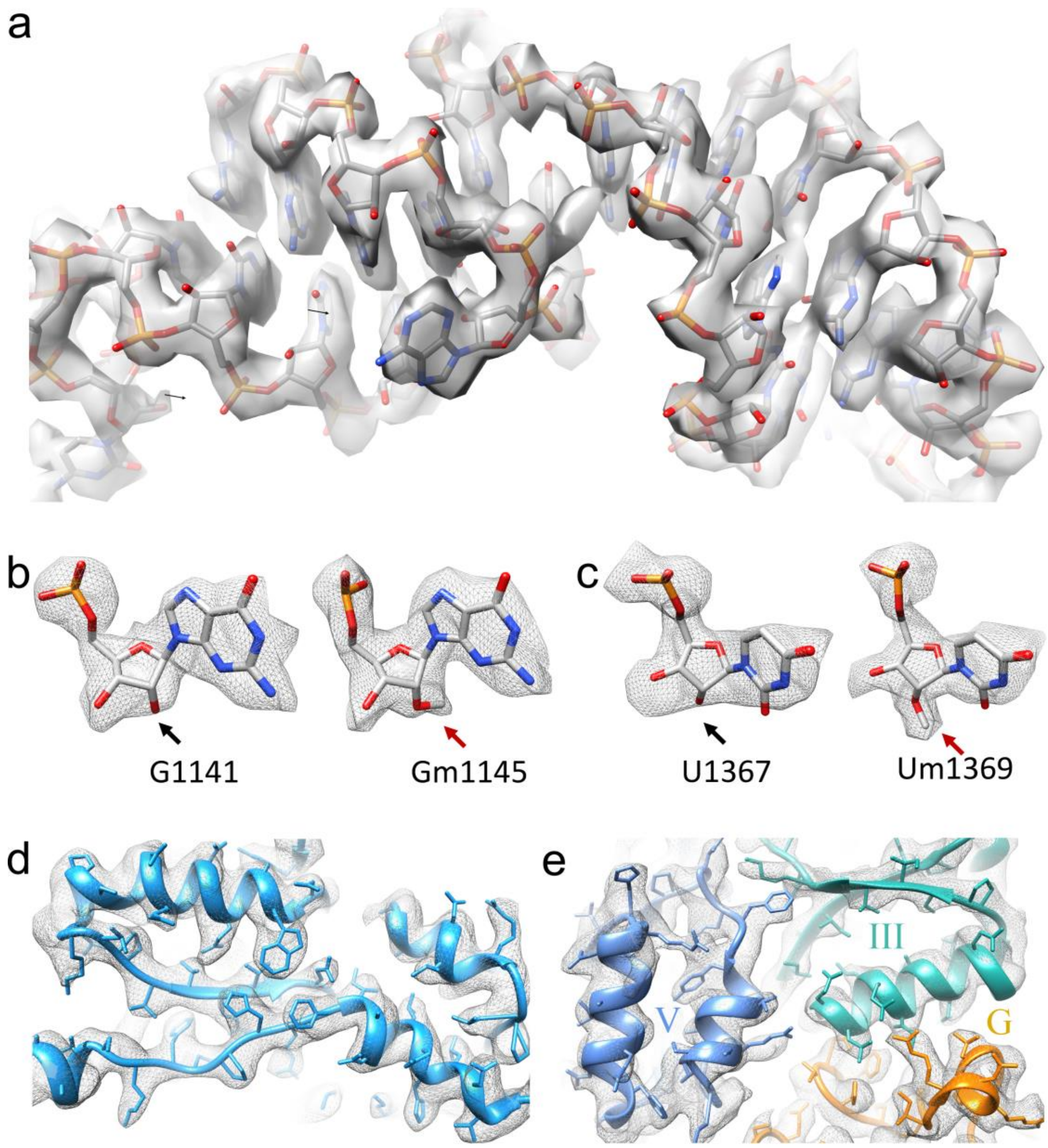

Supplementary Figure 3. Representative segments of cryo-EM densities showing high-resolution features in the Class III 55S•EF-G1$_{mt}$•GMPPCP complex. (**a**) Randomly selected rRNA segment from the 16S rRNA within the 39S LSU. (**b, c**) Show examples of densities corresponding to readily identifiable conserved rRNA methylation sites in nts G1145 and U1369. Densities corresponding to neighboring nts G1141 and U1367 are shown for a side-by-side comparison. (**d, e**) Randomly picked regions from a 39S MRP and EF-G1$_{mt}$, respectively.

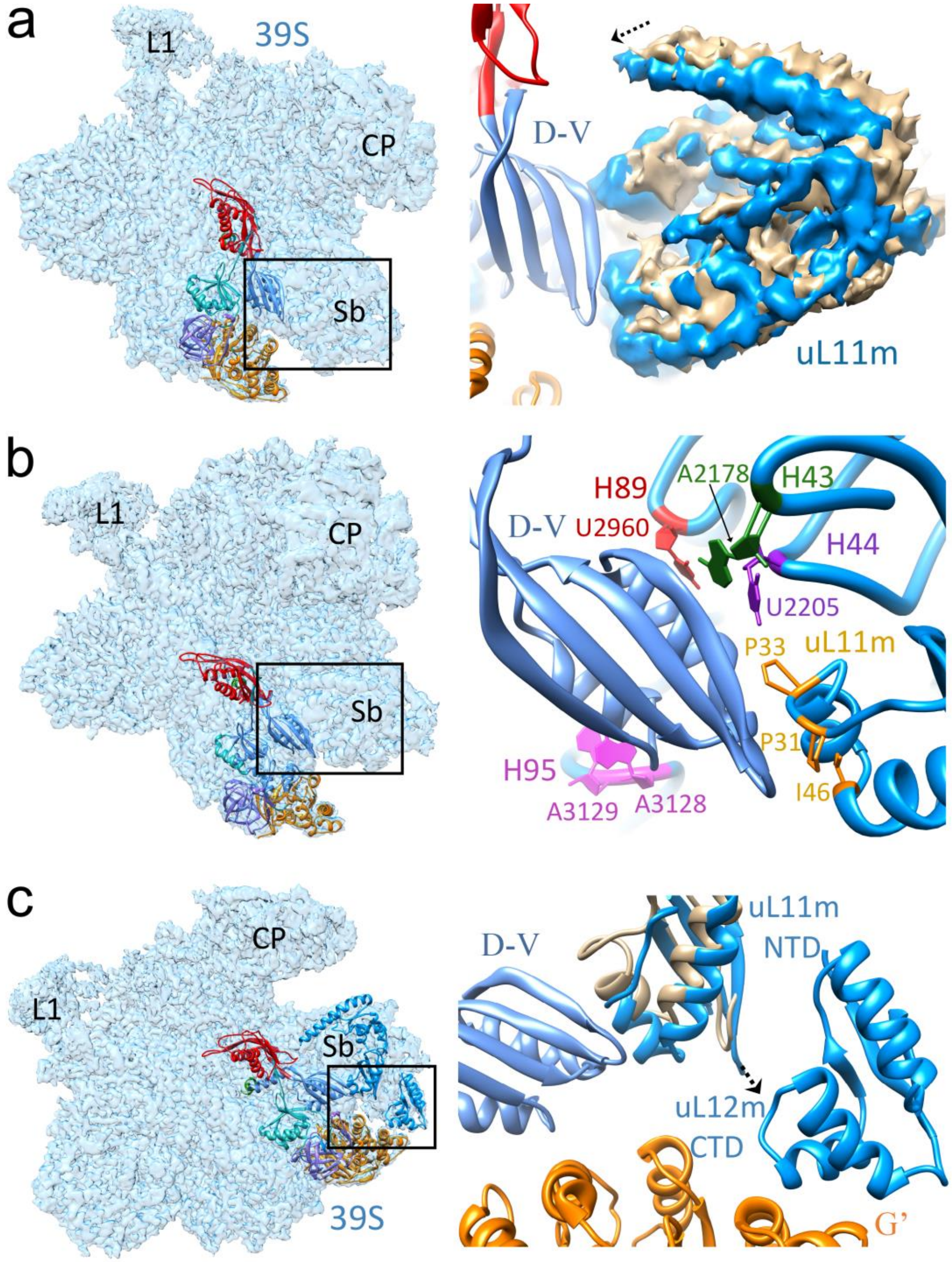

Supplementary Figure 4. Interactions of EF-G1$_{mt}$ domains V and G' with the uL11m and uL12m CTD, respectively. (**a**) The uL11m region (blue) has moved by ~5 Å towards domain V of EF-G1$_{mt}$ in our maps as compared to its position in the vacant human mitoribosome (light brown).[4] (**b**) Domain V provide major anchoring points for the stable binding of EF-G1$_{mt}$ by simultaneously interacting with mitochondrial 16S rRNA residues from helices, H43 (green),

H44 (purple), H89 (red), H95 (pink) and the NTD of uL11m (orange). (**c**) Interactions of the CTD of uL12m. Compared to its position in the vacant human mitoribosome (light brown)[4], the NTD of uL11m (blue) in the 55S•EF-G1$_{mt}$•GMPPCP complex has also moved by ~5 Å towards the CTD of uL12m, allowing simultaneous interactions of CTD of uL12m with both G' subdomain of EF-G1$_{mt}$ (orange) and NTD of uL11m. Thumbnails to the left show the 39S subunit (semitransparent blue) with highlighted positions of EF-G1$_{mt}$, uL11m and CTD of uL12m. Landmarks on the thumbnail: CP, central protuberance; L1, uL1m stalk; and Sb, uL11m stalk base.

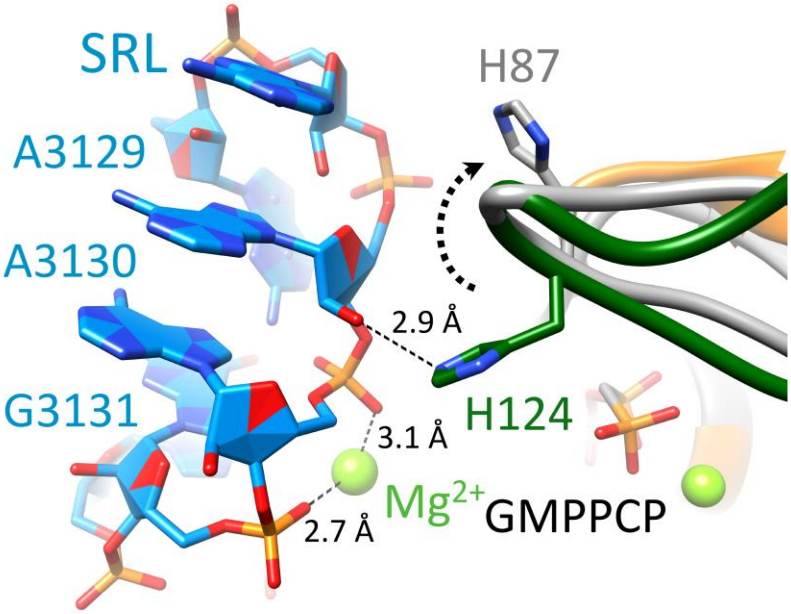

Supplementary Figure 5. Position of the catalytic histidine residue (H124) within the GTP-binding pocket. H124 from the switch II region (dark green) of EF-G1$_{mt}$ is oriented towards the γ phosphate bond of GTP (GMPPCP molecule in our map) while its analogue H87 (gray) is positioned in an opposite direction in the bacterial post-translocational complex in the GDP state.[10] The present active conformation of H124 is stabilized through interactions from base A3129 from the SRL (blue) that are coordinated by the readily visible Mg^{2+} ions (green spheres).

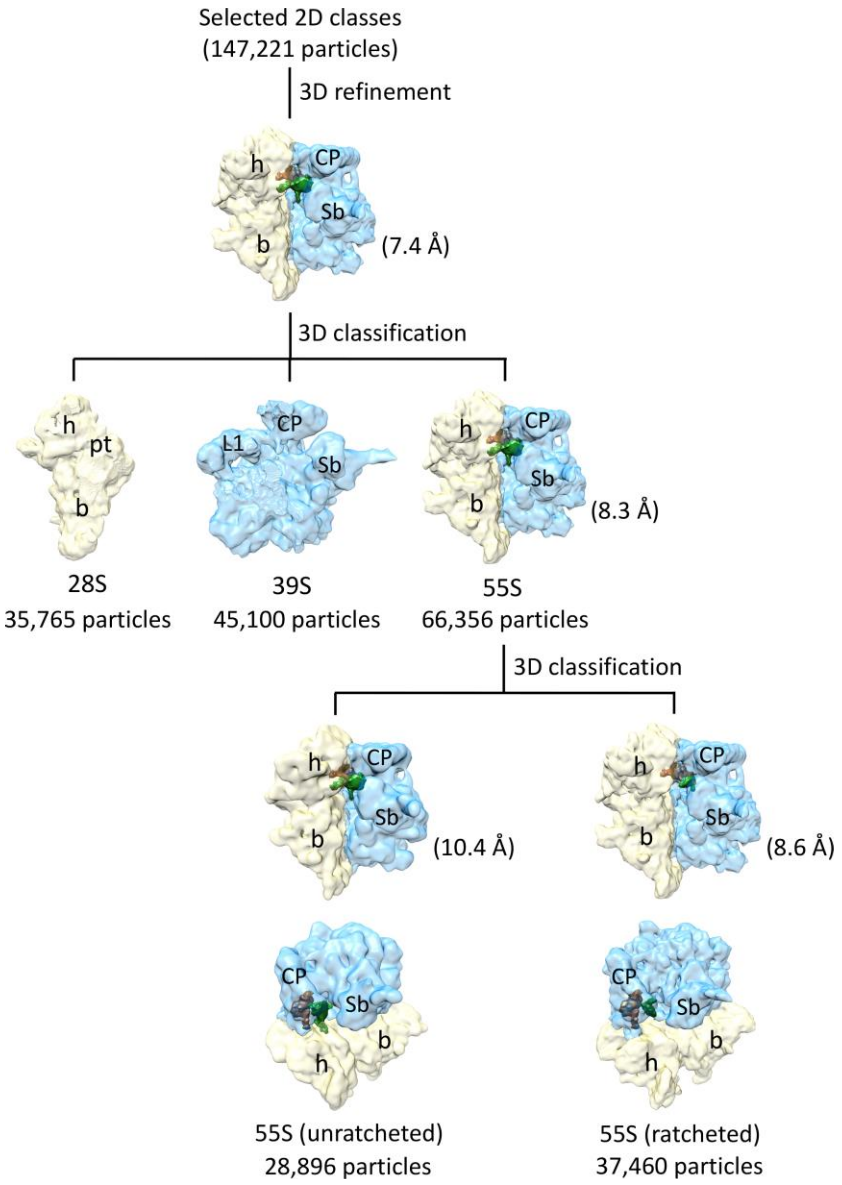

Supplementary Figure 6. Processing of the control human 55S cryo-EM dataset reveals two distinct populations. The flow chart illustrates the main image -processing steps, and the segmented densities of the human mitoribosomal subunits and bound tRNAs. Using reference-based 3D classification, first the intact 55S mitoribosomes (66,356 particles) were separated from the 39S subunits (45,100 particles) and the 28S subunits (37,765 particles). Reclassification of the 55S population yielded two distinct conformational states, with different orientations of the 28S subunit relative to the 39S subunit, and different distributions of the P (green)- and E (brown)-site tRNAs. These two classes were refined to 10.4 Å (unratcheted state) and 8.6 Å (partially ratcheted state).

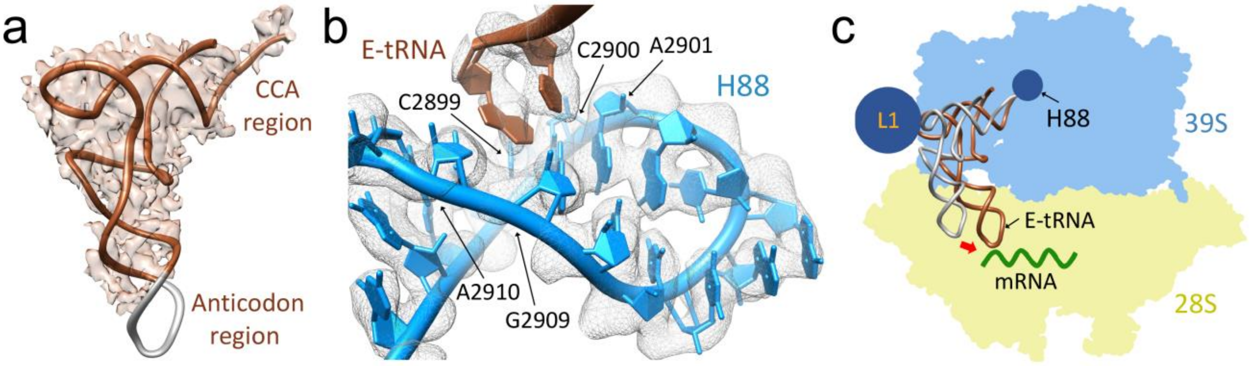

Supplementary Figure 7. Dynamics of tRNA$_{mt}$ in the E site. (**a**) Density corresponding to the E-site tRNA extracted from the Class III complex shows a truncated anticodon region, which is also the case in Class I and Class II complexes. The unmodeled anticodon region of tRNA$_{mt}$ is shown in gray. (**b**) The CCA end of the E-site tRNA$_{mt}$ (brown) is tightly held in position through multiple contacts with 16S rRNA helix, H88 (blue), as also shown by Amunts and coworkers.[4] (**c**) Schematic depicting simultaneous interactions of a regular-sized E-site bound tRNA$_{mt}^{Phe}$ (brown) with H88, uL1m and mRNA. When a smaller sized tRNA$_{mt}$, such as tRNA$_{mt}^{Ser(AGY)}$ (gray) is positioned in the E site, its contacts with all three components simultaneously may not be feasible. If its CCA end and elbow regions are firmly held by H88 and uL1m, respectively, interaction of its anticodon with the mRNA would be disrupted. This would make the density for anticodon arm of the tRNA$_{mt}$ appear weak in the averaged cryo-EM density corresponding to an ensemble of multiple-sized tRNAs$_{mt}$, as observed in panel (a).

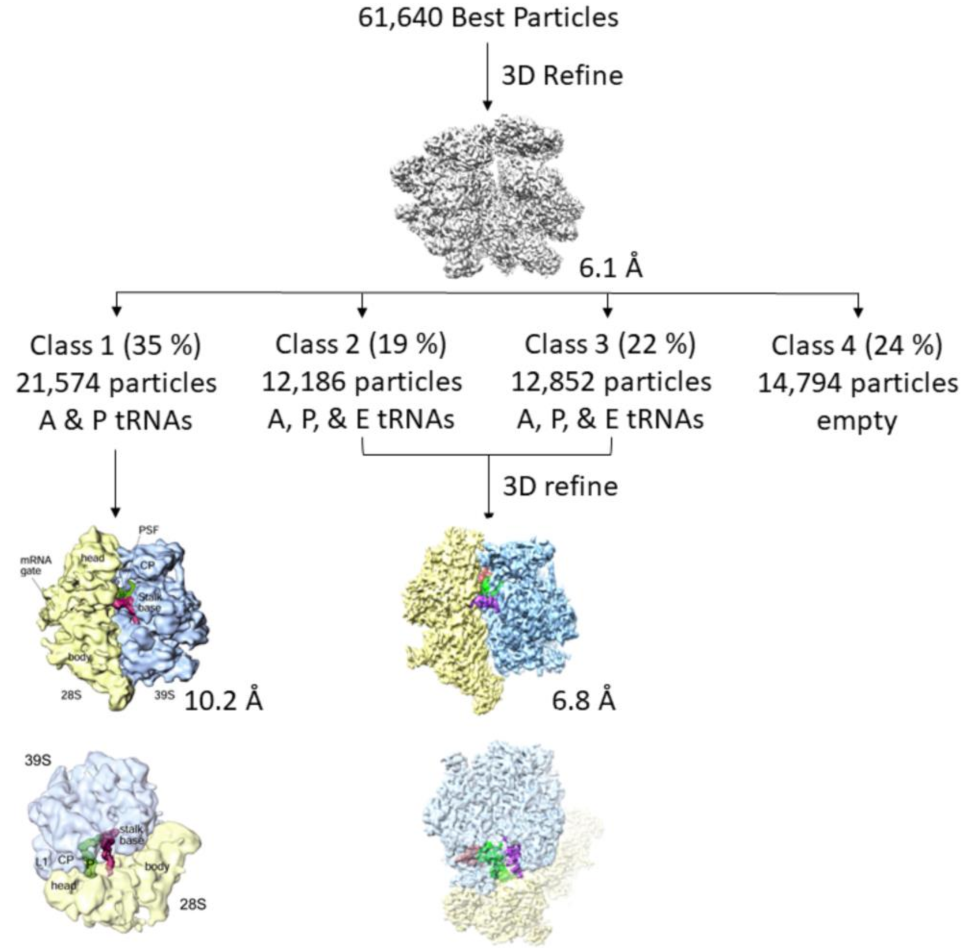

Supplementary Figure 8. Processing of the bovine 55S mitoribosome cryo-EM dataset reveals three distinct populations. The segmented densities of the bovine mitoribosomal subunits and tRNAs are shown in last two rows. Class 1 contains only A (pink) - and P (green)-site tRNAs; Class 2 and 3 carry additional density (fragmented) for the E (brown)-site tRNA, and Class 4 was found to be without any tRNA. In the upper panel the mitoribosome is shown from the 28S shoulder-side and 39S stalk-side view, and in the lower panel it is shown in top views, with 28S subunit (semitransparent yellow) below the 39S subunit (semitransparent blue). Two slightly different orientations of the mitoribosome are used in the lowermost panel to best visualize the tRNAs. These results further validated our previous observation[5,13,17] that the E-site occupancy in bovine ribosome is extremely weak (only ~40 % of the population was found to carry fragmented E-site tRNA density) as compared to that in human mitoribosome. These results also suggest that the occupancies of tRNAs in tissue-derived[5,17,20], and this figure) and cell culture-derived mitoribosomes[4,23]; Figures 1 and 3; Supplementary Figure 6) significantly vary.

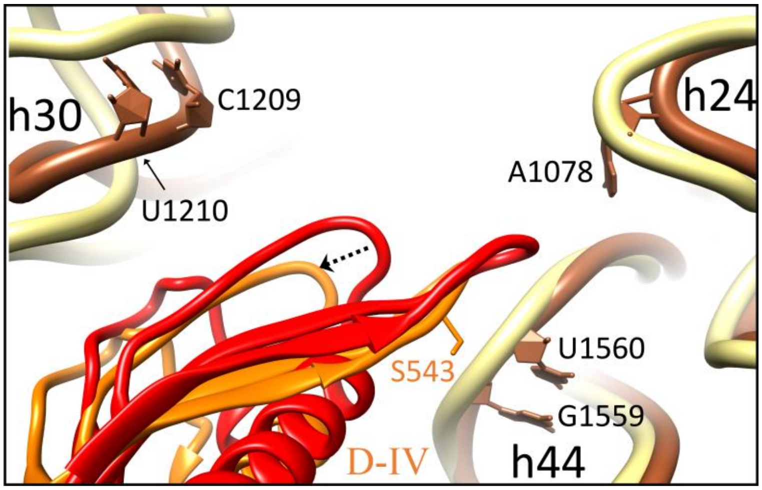

Supplementary Figure 9. Relative position of EF-G1$_{mt}$ domain IV with respect to 28S subunit components in Class I and Class III complexes. In the Class I complex, domain IV (orange) is positioned ~10 Å away from 12S rRNA helices (brown) h30 and h24. In Class III complex, these rRNA helices (yellow) maintain multiple contacts with domain IV (red) (Figure 2i).

```
EF-G1mt    ----MRLL-------------------------GAAAVAALGRG--RAPASLGWQRKQVN   29
EF-G2mt    MLTNLRIFAMSHQTIPSVYINNICCYKIRASLKRLKPHVPLGRNCSSLPGLIGNDI----   56

                                     D-I
EF-G1mt    WKACRWSSSGVIPNEKIRNIGISAHIDSGKTTLTERVLYYTGRIAKMHEVKGKDGVGAVM   89
EF-G2mt    ---KSLHSIINPPIAKIRNIGIMAHIDAGKTTTTERILYYSGYTRSLGDVDDGD---TVT  110

                                     D-I
EF-G1mt    DSMELERQRGITIQSAATYTMWKDVNINIIDTPGHVDFTIEVERALRVLDGAVLVLCAVG  149
EF-G2mt    DFMAQERERGITIQSAAVTFDWKGYRVNLIDTPGHVDFTLEVERCLRVLDGAVAVFDASA  170

                                     D-I
EF-G1mt    GVQCQTMTVNRQMKRYNVPFLTFINKLDRMGSNPARALQQMRSKLNHNAAFMQIPMGLEG  209
EF-G2mt    GVEAQTLTVWRQADKHNIPRICFLNKMDKTGASFKYAVESIREKLKAKPLLLQLPIGEAK  230

                                     D-I
EF-G1mt    NFKGIVDLIEERAIYFDGDFGQIVRYGEI------PAELRAAATDHRQELIECVANSDEQ  263
EF-G2mt    TFKGVVDVVMKEKLLWNCNSNDGKDFERKPLLEMNDPELLKETTEARNALIEQVADLDDE  290

                                     D-I
EF-G1mt    LGEMFLEEKIP-----SISDLKLAIRRATLKRSFTPVFLGSALKNKGVQPLLDAVLEYLP  318
EF-G2mt    FADLVLEEFSENFDLLPAEKLQTAIHRVTLAQTAVPVLCGSALKNKGIQPLLDAVTMYLP  350

                                     D-II
EF-G1mt    NPSEVQNYAILNKEDDSKEKTKILMNSSRDNSHPFVGLAFKL-EVGRFGQLTYVRSYQGE  377
EF-G2mt    SPEERN-YEFLQWYKDD-----------------LCALAFKVLHDKQRGPLVFMRIYSGT  392

                                     D-II
EF-G1mt    LKKGDTIYNTRTRKKVRLQRLARMHADMMEDVEEVYAGDICALFGIDCA-SGDTFT----  432
EF-G2mt    IKPQLAIHNINGNCTERISRLLLPFADQHVEIPSLTAGNIALTVGLKHTATGDTIVSSKS  452

                                                  D-III
EF-G1mt    -------------------DKANSGLSMESIHVPDPVISIAMKPSNKNDLEKFSKGIGRF  473
EF-G2mt    SALAAARRAEREGEKKHRQNNEAERLLLAGVEIPEPVFFCTIEPPSLSKQPDLEHALKCL  512

                                     D-III
EF-G1mt    TREDPTFKVYFDTENKETVISGMGELHLEIYAQRLEREYGCPCITGKPKVAFRETITAPV  533
EF-G2mt    QREDPSLKVRLDPDSGQTVLCGMGELHIEIIHDRIKREYGLETYLGPLQVAYRETILNSV  572

                                     D-IV
EF-G1mt    PFDFTHKKQSGGAGQYGKVIGVLEPLDPEDY---TKLEFSDETFGSNIPKQFVPAVEKGF  590
EF-G2mt    RATDTLDRTLGDKRHLVTVEVEARPIETSSVMPVIEFEYA-ESINEGLLKVSQEAIENGI  631

                                     D-IV
EF-G1mt    LDACEKGPLSGHKLSGLRFVLQDGAHHMVDSNEISFIRAGEGALKQALANATLCILEPIM  650
EF-G2mt    HSACLQGPLLGSPIQDVAITLHSLTIHPGTSTTM-ISACVSRCVQKALKKADKQVLEPLM  690

                                     D-V
EF-G1mt    AVEVVAPNEFQGQVIAGINRRHGVITGQDGVEDYFTLYADVPLNDMFGYSTELRSCTEGK  710
EF-G2mt    NLEVTVARDYLSPVLADLAQRRGNIQEIQTRQDNKVVIGFVPLAEIMGYSTVLRTLTSGS  750

                         D-IV          CTE
EF-G1mt    GEYTMEYSRYQPCLPSTQEDVINKYLEATGQLPVKKGKAKN   751
EF-G2mt    ATFALELSTYQAMNPQDQNTLLNRRSGLT------------   779
```

Supplementary Figure 10. Amino-acid sequence alignment between the human mitochondrial EF-G1$_{mt}$ and EF-G2$_{mt}$. Sequence corresponding to different domains in EF-G1$_{mt}$ are labelled and color-coded as in Figure 1. The crucial glutamic acid residue (E562) that is involved in the interaction of EF-G1$_{mt}$ domain IV with the mito-specific element of uL11m is highlighted in cyan.

a

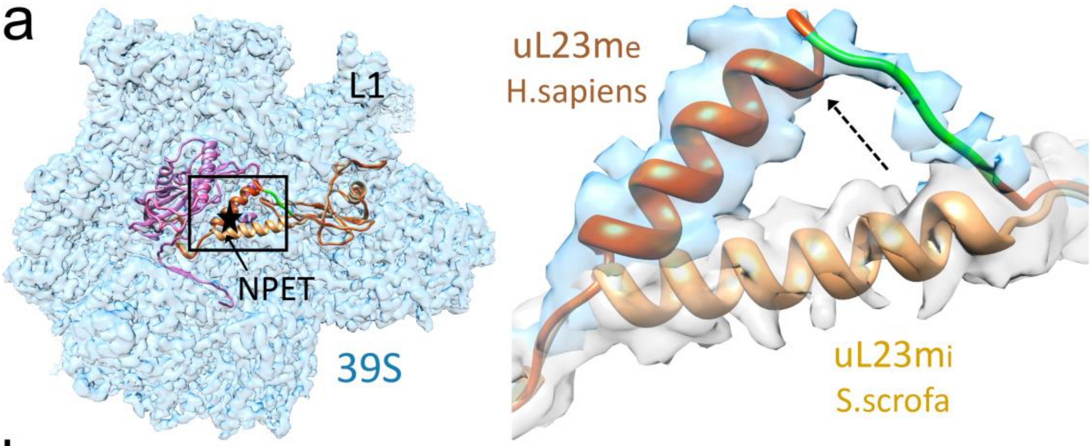

b

```
H.sapiens    MARNVVYPLYRLGGPQLRVFRTNFFIQLVRPGVAQPEDTVQFRIPMEMTRVDLRNYLEGI  60
B.taurus     MARNVLYPLYQLGNPQLRVFRTNFFIQLVRPGTAQPEDTVQFRIPMEMTRVDLRNYLERV  60
S.scrofa     MARNVLYPLYQLGNPQLRVFRTNFFIQLVRPGTAQPEDTVQFRIPMEMTRVDLRNYLERI  60

H.sapiens    YNVPVAAVRTRVQHGSNKRRDHRNVRIKKPDYKVAYVQLAHGQTFTFPDLFPEKDESPEG  120
B.taurus     YNVPVAAVRTRVQHGSNRRRDHRNVRIKQPDYKVAYVQLAHGQTFTFPDLFPKGKRPGGS  120
S.scrofa     YNVPVAAVRTRVQYGSNRRRDHRNIRIKKPDYKVAYVQLALGQTFTFPDLFPERK---GA  117

H.sapiens    SAADDLYSMLEEERQQRQSSDPRRGGVPSWFGL  153
B.taurus     SADEDLRDQVLEEQQQRQSPDPRRGGVPSWFGL  153
S.scrofa     SVDVDVRDQVLEDQRQKHSPDPRRGGVPGWFGL  150
```

Supplementary Figure 11. Large conformational change in the C-terminal region of uL23m. (**a**) Near the exit point of NPET, substantial conformational changes have been observed between the mammalian mitochondrial initiation (light orange)[24] and elongation (orange, current structure) complexes. In addition to the conformational changes corresponding to proteins mL45 (pink) and uL24m described in main text (Figure 6), the C-terminal regions of uL23m also appears to have undergone a large positional change. uL23mi and uL23me, refer to structure of the protein in the initiation and elongation complexes, respectively. (**b**) Alignment of uL23m from human, porcine and bovine mitoribosomes suggests that the region of uL23m that is found to be structurally different in the initiation and elongation complexes might be also due to species related sequence differences (highlighted in green in both panels). Moreover, the α-helix and the associated loop of the C-terminal region of uL23m were poorly resolved in all previous high-resolution structures[4,20,24], making it difficult to draw a functional relevance of observed conformational change in uL23m.

Supplementary Table 1. Data collection, refinement and model validation.

Description	55S•EF-G1$_{mt}$ (Class I)	55S•EF-G1$_{mt}$ (Class III)
**Data collection and Refinement**		
Microscope	FEI Titan Krios	
Voltage (kV)	300	
Pixel size (Å)	1.096	
Defocus range (µm)	1.0 to 3.0	
Average e^{-} dose per image (e^{-}/Å^{2})	69.2	
Software	cryoSPARC	
Particles (initial)	851,131	
Particles (final)	99,804	150,347
Symmetry	C1	C1
FSC-threshold	0.143	0.143
Resolution (Å)	2.97	2.96
Map-sharpening *B* factor (Å^{2}) overall	53.2	57.6
**RMS deviations**		
Bonds (Å)	0.001	0.001
Angles (°)	0.328	0.329
**Molprobity clashscore**	2.02 (75nd)	2.00 (76th)
Clashscore, all atoms	7.6 (83th)	7.2 (86th)
**Rotamer outliers (%)**	0.86	0.86
**Ramachandran plot**		
Favored (%)	87.71	87.77
Outliers (%)	1.56	1.55
**RNA**		
Correct sugar puckers (%)	99.52	99.17
Angle outliers (%)	0.00	0.00
Bond outliers (%)	0.00	0.00
Good backbone conformations (%)	78.48	78.61
**Model composition**		
RNA bases<br>Protein residues	2,725<br>14,978	2,796<br>14,978
**Accession codes**		
Cryo-EM maps	EMD-21233	EMD-21242
PDB ID	6VLZ	6VMI

Supplementary References

1 Mastronarde, D. N. Automated electron microscope tomography using robust prediction of specimen movements. *J Struct Biol* **152**, 36-51, doi:10.1016/j.jsb.2005.07.007 (2005).

2 Rohou, A. & Grigorieff, N. CTFFIND4: Fast and accurate defocus estimation from electron micrographs. *J Struct Biol* **192**, 216-221, doi:10.1016/j.jsb.2015.08.008 (2015).

3 Punjani, A., Rubinstein, J. L., Fleet, D. J. & Brubaker, M. A. cryoSPARC: algorithms for rapid unsupervised cryo-EM structure determination. *Nat Methods* **14**, 290-296, doi:10.1038/nmeth.4169 (2017).

4 Amunts, A., Brown, A., Toots, J., Scheres, S. H. W. & Ramakrishnan, V. Ribosome. The structure of the human mitochondrial ribosome. *Science* **348**, 95-98, doi:10.1126/science.aaa1193 (2015).

5 Sharma, M. R. *et al.* Structure of the mammalian mitochondrial ribosome reveals an expanded functional role for its component proteins. *Cell* **115**, 97-108 (2003).

6 Li, X. *et al.* Electron counting and beam-induced motion correction enable near-atomic-resolution single-particle cryo-EM. *Nat Methods* **10**, 584-590, doi:10.1038/nmeth.2472 (2013).

7 Scheres, S. H. RELION: implementation of a Bayesian approach to cryo-EM structure determination. *J Struct Biol* **180**, 519-530, doi:10.1016/j.jsb.2012.09.006 (2012).

8 Rosenthal, P. B. & Henderson, R. Optimal determination of particle orientation, absolute hand, and contrast loss in single-particle electron cryomicroscopy. *J Mol Biol* **333**, 721-745, doi:10.1016/j.jmb.2003.07.013 (2003).

9 Agrawal, R. K. *et al.* Visualization of tRNA movements on the Escherichia coli 70S ribosome during the elongation cycle. *J Cell Biol* **150**, 447-460, doi:10.1083/jcb.150.3.447 (2000).

10 Gao, Y. G. *et al.* The structure of the ribosome with elongation factor G trapped in the posttranslocational state. *Science* **326**, 694-699, doi:10.1126/science.1179709 (2009).

11 Lin, J., Gagnon, M. G., Bulkley, D. & Steitz, T. A. Conformational changes of elongation factor G on the ribosome during tRNA translocation. *Cell* **160**, 219-227, doi:10.1016/j.cell.2014.11.049 (2015).

12 Zhou, J., Lancaster, L., Donohue, J. P. & Noller, H. F. Crystal structures of EF-G-ribosome complexes trapped in intermediate states of translocation. *Science* **340**, 1236086, doi:10.1126/science.1236086 (2013).

13 Mears, J. A. *et al.* A structural model for the large subunit of the mammalian mitochondrial ribosome. *J Mol Biol* **358**, 193-212, doi:10.1016/j.jmb.2006.01.094 (2006).

14 Bocchetta, M., Xiong, L., Shah, S. & Mankin, A. S. Interactions between 23S rRNA and tRNA in the ribosomal E site. *RNA* **7**, 54-63, doi:10.1017/s1355838201001650 (2001).

15 Selmer, M. *et al.* Structure of the 70S ribosome complexed with mRNA and tRNA. *Science* **313**, 1935-1942, doi:10.1126/science.1131127 (2006).

16 Yusupov, M. M. *et al.* Crystal structure of the ribosome at 5.5 A resolution. *Science* **292**, 883-896, doi:10.1126/science.1060089 (2001).

17 Kaushal, P. S., Sharma, M. R. & Agrawal, R. K. The 55S mammalian mitochondrial ribosome and its tRNA-exit region. *Biochimie* **114**, 119-126, doi:10.1016/j.biochi.2015.03.013 (2015).

18 Ramrath, D. J. *et al.* Visualization of two transfer RNAs trapped in transit during elongation factor G-mediated translocation. *Proc Natl Acad Sci U S A* **110**, 20964-20969, doi:10.1073/pnas.1320387110 (2013).
19 Schuwirth, B. S. *et al.* Structures of the bacterial ribosome at 3.5 A resolution. *Science* **310**, 827-834, doi:10.1126/science.1117230 (2005).
20 Greber, B. J. *et al.* Ribosome. The complete structure of the 55S mammalian mitochondrial ribosome. *Science* **348**, 303-308, doi:10.1126/science.aaa3872 (2015).
21 Kaushal, P. S. *et al.* Cryo-EM structure of the small subunit of the mammalian mitochondrial ribosome. *Proc Natl Acad Sci U S A* **111**, 7284-7289, doi:10.1073/pnas.1401657111 (2014).
22 Koripella, R. K. *et al.* Structure of Human Mitochondrial Translation Initiation Factor 3 Bound to the Small Ribosomal Subunit. *iScience* **12**, 76-86, doi:10.1016/j.isci.2018.12.030 (2019).
23 Koripella, R. K., Sharma, M. R., Risteff, P., Keshavan, P. & Agrawal, R. K. Structural insights into unique features of the human mitochondrial ribosome recycling. *Proc Natl Acad Sci U S A* **116**, 8283-8288, doi:10.1073/pnas.1815675116 (2019).
24 Kummer, E. *et al.* Unique features of mammalian mitochondrial translation initiation revealed by cryo-EM. *Nature* **560**, 263-267, doi:10.1038/s41586-018-0373-y (2018).
