# Supplementary material for: Structures of the human mitochondrial ribosome bound to EF-G1 reveal distinct features of mitochondrial translation elongation
Source: Nat Commun. 2020 Jul 31;11:3830. doi: 10.1038/s41467-020-17715-2 (PMC7395135; doi:10.1038/s41467-020-17715-2)
Supplement: Supplementary file 3 — Reporting Summary [file 41467_2020_17715_MOESM3_ESM.pdf]

nature research

Corresponding author(s): Rajendra K. Agrawal

Last updated by author(s): Apr 15, 2020

Reporting Summary

Nature Research wishes to improve the reproducibility of the work that we publish. This form provides structure for consistency and transparency in reporting. For further information on Nature Research policies, see Authors & Referees and the Editorial Policy Checklist .

Statistics

For all statistical analyses, confirm that the following items are present in the figure legend, table legend, main text, or Methods section.

n/a	Confirmed	
☒	☐	The exact sample size (*n*) for each experimental group/condition, given as a discrete number and unit of measurement
☒	☐	A statement on whether measurements were taken from distinct samples or whether the same sample was measured repeatedly
☒	☐	The statistical test(s) used AND whether they are one- or two-sided<br>*Only common tests should be described solely by name; describe more complex techniques in the Methods section.*
☒	☐	A description of all covariates tested
☒	☐	A description of any assumptions or corrections, such as tests of normality and adjustment for multiple comparisons
☒	☐	A full description of the statistical parameters including central tendency (e.g. means) or other basic estimates (e.g. regression coefficient) AND variation (e.g. standard deviation) or associated estimates of uncertainty (e.g. confidence intervals)
☒	☐	For null hypothesis testing, the test statistic (e.g. *F*, *t*, *r*) with confidence intervals, effect sizes, degrees of freedom and *P* value noted<br>*Give P values as exact values whenever suitable.*
☒	☐	For Bayesian analysis, information on the choice of priors and Markov chain Monte Carlo settings
☒	☐	For hierarchical and complex designs, identification of the appropriate level for tests and full reporting of outcomes
☒	☐	Estimates of effect sizes (e.g. Cohen's *d*, Pearson's *r*), indicating how they were calculated

Our web collection on statistics for biologists contains articles on many of the points above.

Software and code

Policy information about availability of computer code

Data collection	Leginon (emg.nysbc.org/redmine/projects/leginon) automated data collection on a Titan Krios electron microscope (ThermoFisher)
Data analysis	CryoSPARC version 2.14, Relion 2.0, UCSF Chimera 1.14, COOT, and PHENIX.

For manuscripts utilizing custom algorithms or software that are central to the research but not yet described in published literature, software must be made available to editors/reviewers. We strongly encourage code deposition in a community repository (e.g. GitHub). See the Nature Research guidelines for submitting code & software for further information.

Data

Policy information about availability of data

All manuscripts must include a data availability statement. This statement should provide the following information, where applicable:

- Accession codes, unique identifiers, or web links for publicly available datasets
- A list of figures that have associated raw data
- A description of any restrictions on data availability

Data Deposition: The cryo-EM maps and atomic coordinates have been deposited in the Electron Microscopy and PDB Data Bank (wwPDB.org) under accession codes EMD-21233 and PDB ID: 6VLZ for the EF-G1mt-bound 55S mitoribosome (Complex I), and EMD-21242 and PDB ID: 6VMI for the EF-G1mt-bound 55S mitoribosome (Complex III). Cryo-EM maps of the Complex II and bovine 55S mitoribosome have been deposited with accession codes EMD-22212 and EMD-22209, respectively.

All raw structural data is being made available through empiar, an electron microscopy public image archive.

Field-specific reporting

Please select the one below that is the best fit for your research. If you are not sure, read the appropriate sections before making your selection.

- [x] Life sciences
- [ ] Behavioural & social sciences
- [ ] Ecological, evolutionary & environmental sciences

For a reference copy of the document with all sections, see nature.com/documents/nr-reporting-summary-flat.pdf

Life sciences study design

All studies must disclose on these points even when the disclosure is negative.

Sample size	Biological sample preparation was performed in vitro, using purified mitochondrial ribosomes from a human cell line and an over-expressed translational factor EF-G1. After grid preparation and cryo-EM data collection, 1.61 million single particle images derived from 6,671 electron micrographs were collected. About 50 % of them (851,131 particle images) were retained after screening and used in image processing. The retained dataset was further classified to obtain structurally stable groups.
Data exclusions	Particles images representing dissociated ribosomal subunits were excluded from the final analyses.
Replication	The single particle dataset was randomly divided and processed, each time giving reproducible results, consistent with the results presented in the paper.
Randomization	The single particle data processing involved dataset randomization for consistency.
Blinding	Two authors have independently processed the dataset to arrive at the same results and conclusions.

Reporting for specific materials, systems and methods

We require information from authors about some types of materials, experimental systems and methods used in many studies. Here, indicate whether each material, system or method listed is relevant to your study. If you are not sure if a list item applies to your research, read the appropriate section before selecting a response.

Materials & experimental systems

n/a	Involved in the study
☒	☐ Antibodies
☐	☒ Eukaryotic cell lines
☒	☐ Palaeontology
☒	☐ Animals and other organisms
☒	☐ Human research participants
☒	☐ Clinical data

Methods

n/a	Involved in the study
☒	☐ ChIP-seq
☒	☐ Flow cytometry
☒	☐ MRI-based neuroimaging

Eukaryotic cell lines

Policy information about cell lines

Cell line source(s)	HEK293S GnTI Cell were originally obtained from the Har Gobind Khorana lab at MIT.
Authentication	Cell lines were not further authenticated for this study.
Mycoplasma contamination	Cell lines were further tested for contamination by mycoplasma.
Commonly misidentified lines (See ICLAC register)	No commonly misidentified cell lines were used in this work.
