# Supplementary material for: Structures of the human mitochondrial ribosome bound to EF-G1 reveal distinct features of mitochondrial translation elongation
Source: Nat Commun. 2020 Jul 31;11:3830. doi: 10.1038/s41467-020-17715-2 (PMC7395135; doi:10.1038/s41467-020-17715-2)
Supplement: Supplementary file 2 — Peer Review [file 41467_2020_17715_MOESM2_ESM.pdf]

Reviewer #1 (Remarks to the Author):

While there are many structures of mitochondrial ribosomes, the majority have been of isolated subunits or vacant ribosomes. Very few have reported defined functional states and insights into mitochondrial translocation catalyzed by EF-G1 have been lacking. The manuscript of Koripella and coworkers reports structures of human mitochondrial ribosomes bound with EFG reporting insights into mitochondrial translation elongation. Specifically, three structures are presented with EF-G, P-site and E-site tRNAs with differing degrees of ratcheting and head swivel. The resolution of two major states is excellent (3A) whereas the minor state is less-well resolved (4A). Comparison of these states in the light of the wealth of knowledge from bacterial ribosome structures with EF-G, sheds insights into how mitochondrial EF-G1 interacts with the ribosome and could facilitate translocation of tRNAs. While many of the conclusions are consistent and similar to that observed previously on bacterial ribosomes, a collection of novel insights due to mitochondrial specific elements are also revealed. Thus, the study will be of interest to researcher working in the field of translation. However, there are a few major points that need to be addressed that would significantly increase the significance of the paper as well as some minor ones that should be addressed to improve the clarity and accuracy of the manuscript.

Major points:

1. It remains unclear as to the functional state that is being analyzed here i.e. was this a post-state ribosome to which EF-G bound or was the starting point a PRE state to which EF-G bound and initiated translocation. The latter would be physiologically more relevant and interesting. Therefore, I am surprised that the authors have not clarified this. One would have expected that not all ribosome particles would have bound EF-G so that unbound ribosomes could be analyzed and the state determined as PRE or POST. However, surprisingly, from Fig S1, it seems that all 55S had EF-G whereas the rest were split into subunits? In this case, it would seem appropriate to make a small data collection in the absence of EF-G to verify the starting state – this does not been to be on a high-end microscope, nor have high resolution, low resolution (10-15A) that one can attain even on Talos 120kV TEM would be sufficient to address this point.

2. Related to point 1 with respect to the funcational state. The authors suggest that their results provide insights into translation elongation and their ribosomes support the suggestion since they carry a mixture of tRNAs. However, what is surprising therefore is that there is apparantly no nascent polypeptide chain? Even with a mixture of different nascent chains, one can low pass filter the maps and observe density for nascent chains through the tunnel. Is this the case? If not, why not? Has the nascent chain been hydrolyzed during purification? If the authors want to put the movements of ribosomal proteins within the NPET within the context of translation elongation (rather than initiation), then they need to address this issue.

3. Page 13, It seems that the authors plan to deposit only maps and models for Class I and III. This is not appropriate, the authors should deposit at least the map for Class II also. Also maps for the Bovine mitoribosome since they are presented here as well?

Minor points:

Fig. 1; I find it strange not to show an overview also for class I and II…with the exception of a rotation view in Fig 2, these overviews can only be found in small insets in Fig S1 but the tRNAs cannot be seen here.

Fig. S1. Perhaps its pertinent to point out that the resolutions are calculated based on the FSC 0.143 criteria? Its not even mentioned in the text what criteria are used. Associated references would seem appropriate, especially given that Agrawal and Frank were for along time using the FSC 0.5 criteria and not the FSC 0.143!

Fig S1. The resolution of Class I and III is 3A. What is the resolution of the multibody-refined small and large subunit and EF-G…did it improve significantly like it should. The average resolution could also be indicated on this figure.

Fig S2…it appears that the authors provide only a local resolution of the locally refined EF-G from all particles in the dataset, rather than also showing the local resolution of EF-G in Class I, II and III. In fact, the authors should really also show the local resoluton for the A and P-tRNAs in each class. Its important to understand the resolution of the ligands in each class so as to ascertain whether the interpretation and level of details is possible. Its clear that the ribosome is well-resolved as always, but even here its unclear exactly what c and d depict…overviews, transverse sections, which views? There are also no landmarks indicated.

Fig 1h. The dashed lines showing hydrogen bonds or ion coordination are apparantly drawn using illustrator rather than being generated in the visualization software since the lines often are not going to the center of the atoms/ions. Moreover, in some cases the distance and/or geometries do not appear to be optimal/possible to facilitate such as an interaction. Maybe the authors want to double check them and perhaps also provide the distances, especially for the H124 and D56 contacts.

Fig. 2 maybe some color codes in the figure to help the reader, especially when the legends are unclear…what is (c)? presumably (c) is class III? Should it not be shown first as panel (a) and then have (b) and (c) which would be comparisons with class I and II, respectively?

Reviewer #2 (Remarks to the Author):

The 'Structures of the human mitochondrial ribosome bound to EF-G1......' by Koripella et al., is a beautiful piece of work, where cryo-EM has been used in the best way to present a complete picture of mRNA-tRNA translocation in human mitoribosomes. The illustrations are appropriate and backed up with strong supplementary figures. The combined results and discussions answer almost all the questions I could pose regarding translocation in mitochondria. The discussions of the roles of the mitoribosome specific features, specially in relation to the interaction with P- and E-site tRNAs, and the different configurations of the mL45 with respect to the NPET element in the initiation and elongation complexes, make the study very strong. The paper can be recommended for immediate publication. However, I feel that the discussion will be more enriched if the authors can discuss in the light of their EF-G1 structure, and comparison of its sequence with that of EF-G2, why mitochondrial EF-G2 cannot translocate while EF-G1 can. I also wonder which biochemical experiments are meant in this study as mentioned in the author contributions. In fact, adding some biochemistry of mitoribosome translocation in this paper would be like a cherry on the cake.

RESPONSE TO REVIEWERS' COMMENTS

Reviewer #1:

While there are many structures of mitochondrial ribosomes, the majority have been of isolated subunits or vacant ribosomes. Very few have reported defined functional states and insights into mitochondrial translocation catalyzed by EF-G1 have been lacking. The manuscript of Koripella and coworkers reports structures of human mitochondrial ribosomes bound with EFG reporting insights into mitochondrial translation elongation. Specifically, three structures are presented with EF-G, P-site and E-site tRNAs with differing degrees of ratcheting and head swivel. The resolution of two major states is excellent (3A) whereas the minor state is less-well resolved (4A). Comparison of these states in the light of the wealth of knowledge from bacterial ribosome structures with EF-G, sheds insights into how mitochondrial EF-G1 interacts with the ribosome and could facilitate translocation of tRNAs. While many of the conclusions are consistent and similar to that observed previously on bacterial ribosomes, a collection of novel insights due to mitochondrial specific elements are also revealed. Thus, the study will be of interest to researcher working in the field of translation. However, there are a few major points that need to be addressed that would significantly increase the significance of the paper as well as some minor ones that should be addressed to improve the clarity and accuracy of the manuscript.

Response: We thank the reviewer for constructive comments that have helped us improve the overall quality of the paper.

Major points:

Comment 1: *It remains unclear as to the functional state that is being analyzed here i.e. was this a post-state ribosome to which EF-G bound or was the starting point a PRE state to which EF-G bound and initiated translocation. The latter would be physiologically more relevant and interesting. Therefore, I am surprised that the authors have not clarified this. One would have expected that not all ribosome particles would have bound EF-G so that unbound ribosomes could be analyzed and the state determined as PRE or POST. However, surprisingly, from Fig S1, it seems that all 55S had EF-G whereas the rest were split into subunits? In this case, it would seem appropriate to make a small data collection in the absence of EF-G to verify the starting state – this does not been to be on a high-end microscope, nor have high resolution, low resolution (10-15A) that one can attain even on Talos 120kV TEM would be sufficient to address this point.*

Response: We agree with the reviewer about this important point. We had also expected to obtain a population with EF-G1$_{mt}$-unbound mitoribosomes but it was not found, even after employing several round of 3D classifications, including masked local classifications. As suggested, we have now collected new data and include a cryo-EM structural analysis of the same batch of the mitoribosome preparation as a control (Fig. S6). We find that a majority of the human mitoribosomes show tRNA$_{mt}$ density in the E-site region, whereas a fraction of those mitoribosomes also show tRNA$_{mt}$ density in the P-site region. 3D classification shows that the control mitoribosomes exist primarily in two conformational states. The relative abundance of two control conformational states (56 % and 44 %, respectively), suggests that EF-G1-binding brings a greater proportion of mitoribosomes into unratcheted state. A brief discussion in light of the new findings have also been added (page 7, first continued para, lines 3-10)

Comment 2: *Related to point 1 with respect to the funcational state. The authors suggest that their results provide insights into translation elongation and their ribosomes support the suggestion since*

they carry a mixture of tRNAs. However, what is surprising therefore is that there is apparantly no nascent polypeptide chain? Even with a mixture of different nascent chains, one can low pass filter the maps and observe density for nascent chains through the tunnel. Is this the case? If not, why not? Has the nascent chain been hydrolyzed during purification? If the authors want to put the movements of ribosomal proteins within the NPET within the context of translation elongation (rather than initiation), then they need to address this issue.

Response: We concur with this important point. As suggested, we have located a clear density for the nascent polypeptide chain linked to CCA end of the P-site tRNA EF-G1-bound Complex III map, but not in Complex I and II maps, which also have either no or a weak P-site tRNA, respectively. We find the N-terminus of nascent polypeptide chain juxtaposed to the N-terminus of the mL45 and an adenine residue of the 16S rRNA localized within the NPET. This important finding has been added as a new panel (panel a) in Fig. 6, and its possible functional implication has been briefly described in the last paragraph on page 11, continued on to page 12.

Comment 3: *Page 13, It seems that the authors plan to deposit only maps and models for Class I and III. This is not appropriate, the authors should deposit at least the map for Class II also. Also maps for the Bovine mitoribosome since they are presented here as well?*

Response: As suggested, we have now deposited the maps for Class II and bovine mitoribosome (see the updated Data Deposition section on page 14).

Minor points:

Comment: *Fig. 1; I find it strange not to show an overview also for class I and II…with the exception of a rotation view in Fig 2, these overviews can only be found in small insets in Fig S1 but the tRNAs cannot be seen here.*

Response: We have now provided the segmented overviews for the Class I and II maps as well (Fig. 3d-f), and have described the related observation on page 7 (in paragraph continued from page 6).

Comment: *Fig. S1. Perhaps its pertinent to point out that the resolutions are calculated based on the FSC 0.143 criteria? Its not even mentioned in the text what criteria are used. Associated references would seem appropriate, especially given that Agrawal and Frank were for along time using the FSC 0.5 criteria and not the FSC 0.143!*

Response: We have now clarified in the manuscript that the resolutions are calculated according to the gold-standard criterion. All image processings were performed using either cryoSPARC or Relion (for *Bos taurus* control dataset), where the gold-standard criterion using FSC 0.143 has been implemented as default method for resolution calculation, and we reported resolutions of our maps accordingly. An appropriate reference has also been added (Supplementary Information page 3, last line of the first continued para).

Comment: *Fig S1. The resolution of Class I and III is 3A. What is the resolution of the multibody-refined small and large subunit and EF-G…did it improve significantly like it should. The average resolution could also be indicated on this figure.*

Response: We have mentioned average resolution of each reconstruction either next to or below the map in Fig. S1, whereas the results of local refinement and local resolutions are shown in Fig. S2.

Comment: *Fig S2…it appears that the authors provide only a local resolution of the locally refined EF-G from all particles in the dataset, rather than also showing the local resolution of EF-G in Class I, II and III. In fact, the authors should really also show the local resoluton for the A and P-tRNAs in each class. Its important to understand the resolution of the ligands in each class so as to ascertain whether the interpretation and level of details is possible. Its clear that the ribosome is well-resolved as always, but even here its unclear exactly what c and d depict…overviews, transverse sections, which views? There are also no landmarks indicated.*

Response: As mentioned in the text (page 7, last sentence continued onto page 8), the tRNA densities are not fully resolved, likely because they represent an ensemble average of indigenously-bound heterogeneous-sized mitochondrial tRNAs. Moreover, the proportion of tRNA-bound mitoribosomes isolated from human cell lines are relatively low, as compared to that in mammalian mitoribosomes isolated from animal tissues, such as the bovine mitoribosome. This is why we included the bovine data as control for positional reference to A-, P- and E-site tRNAs (Fig. S7). As suggested, we have now provided the local resolution of EF-G1mt in the combined map (Fig. S2b), as well as in three individual classes (Fig. S2c-e). Each of panels c-e show local resolution of the entire 55S complex, and individually refined subunits on the right-hand side in the same row. Densities shown in all panels are surface views (no transverse sections). We have now further clarified this in the Fig. S2 legend title, and have added landmarks of subunit in panels a-c of the same figure.

Comment: *Fig 1h. The dashed lines showing hydrogen bonds or ion coordination are apparantly drawn using illustrator rather than being generated in the visualization software since the lines often are not going to the center of the atoms/ions. Moreover, in some cases the distance and/or geometries do not appear to be optimal/possible to facilitate such as an interaction. Maybe the authors want to double check them and perhaps also provide the distances, especially for the H124 and D56 contacts.*

Response: We have now updated the relevant panels, following the reviewer's suggestion. This further clarified the interactions and led to minor changes in contacts described on page 6 (lines 12 and 14). Also, we have now split the existing Fig 1 into two parts, by moving lower four panels (Fig. 1e-h) as a new Fig. 2. All subsequent figures have been accordingly renumbered.

Comment: *Fig. 2 maybe some color codes in the figure to help the reader, especially when the legends are unclear…what is (c)? presumably (c) is class III? Should it not be shown first as panel (a) and then have (b) and (c) which would be comparisons with class I and II, respectively?*

Response: Fig. 2 is now Fig 3, for the reason mentioned above. To avoid confusion, we have numbered classes according to likely biological sequence of events (see page 9, lines 4-7). Also, by addressing the first minor comment, we addded the segmented overviews of bound ligands and subunits for all three classes (panels d-f). Each of the three classes are now identified in the figure that further clarify the color codes.

<u>Reviewer #2</u>:

The 'Structures of the human mitochondrial ribosome bound to EF-G1......' by Koripella et al., is a beautiful piece of work, where cryo-EM has been used in the best way to present a complete picture of mRNA-tRNA translocation in human mitoribosomes. The illustrations are appropriate and backed up with strong supplementary figures. The combined results and discussions answer almost all the questions I could pose regarding translocation in mitochondria. The discussions of the roles of the mitoribosome specific features, specially in relation to the interaction with P- and E-site tRNAs, and the different configurations of the mL45 with respect to the NPET element in the initiation and elongation complexes, make the study very strong. The paper can be recommended for immediate publication.

Response: We thank the reviewer for these encouraging comments.

Minor points:

Comment: *However, I feel that the discussion will be more enriched if the authors can discuss in the light of their EF-G1 structure, and comparison of its sequence with that of EF-G2, why mitochondrial EF-G2 cannot translocate while EF-G1 can.*

Response: We have now added a comparison of sequences between EF-G1 and EF-G2 (Fig. S10). In doing so, we also made an important observation that a uniquely placed aa residue E562 in EF-G1 is poised to make an interaction with the mito-specific segment of protein uL11m. We have added this observation in form of a new panel "*d*" to the existing Fig 3 (now Fig. 4), and have briefly described the new findings and likely reasons that functionally differentiate the two factors (Page 10, 1st complete para).

Comment: *I also wonder which biochemical experiments are meant in this study as mentioned in the author contributions.*

Response: We have now more specifically itemized the author's contributions (page 14).

Comment: *In fact, adding some biochemistry of mitoribosome translocation in this paper would be like a cherry on the cake.*

Response: We are presently engaged in the process of standardizing these biochemical assays on human mitoribosome translation, which remain challenging due to limited amounts of the mitoribosome and mito-tRNAs that can be purified from human cell lines. The reviewer's suggestion is well taken for future studies, but to add any additional biochemical data would impose unpredictably long delay to the publication of the current results.

REVIEWERS' COMMENTS:

Reviewer #1 (Remarks to the Author):

The authors have made an excellent effort to address all my comments. I would like to thank them for highlighting the changes in the text which makes it easier to find the sections (except for the FSC criteria which they didn't highlight unfortunately).

The addition of the small dataset of the control mitoribosome is definitely an important addition. I would only ask that the authors clearly describe not just the ratcheted or non-ratcheted state but also the conformation of the tRNAs i.e. P/P- and E/E and A/P and P/E, I presume?

I have only one last minor small point which is that the use of 16S rRNA is a little confusing to readers who are not used to mitoribosomes since it is the same size of the 16S rRNA that is in the small subunit of bacteria. Maybe it makes sense to use SSU and LSU rRNA or to have both 12S (SSU) rRNA and 16S (LSU) rRNA, particular in the figures to avoid any confusion.

RESPONSE TO REVIEWERS' COMMENTS

Reviewer #1:

Comment: *The authors have made an excellent effort to address all my comments. I would like to thank them for highlighting the changes in the text which makes it easier to find the sections. The addition of the small dataset of the control mitoribosome is definitely an important addition. I would only ask that the authors clearly describe not just the ratcheted or non-ratcheted state but also the conformation of the tRNAs i.e. P/P- and E/E and A/P and P/E, I presume?*

Response: The tRNA positions in the control human mitoribosome maps are found to be primarily in P/P and E/E states, despite the observed SSU rotation with respect to the LSU in one of the two populations. Moreover, we have further clarified the tRNA states in the EF-G1mt-bound complexes, without affecting the original description.

Comment: *I have only one last minor small point which is that the use of 16S rRNA is a little confusing to readers who are not used to mitoribosomes since it is the same size of the 16S rRNA that is in the small subunit of bacteria. Maybe it makes sense to use SSU and LSU rRNA or to have both 12S (SSU) rRNA and 16S (LSU) rRNA, particular in the figures to avoid any confusion.*

Response: We have now added SSU and LSU along with with 12S and 16S rRNAs, respectively, throughout the text as appropriate. Also, we have labeled the 12S and 16S rRNA in Figure 1b, and the 16S rRNA in Figure 6a.
